# Sensitivity of substrate translocation in chaperone-mediated autophagy to Alzheimer’s disease progression

**DOI:** 10.18632/aging.205856

**Published:** 2024-05-23

**Authors:** Lei Yu, Xinping Pang, Lin Yang, Kunpei Jin, Wenbo Guo, Yanyu Wei, Chaoyang Pang

**Affiliations:** 1 College of Computer Science, Sichuan Normal University, Chengdu 610101, China; 2 West China School of Basic Medical Sciences and Forensic Medicine, Sichuan University, Chengdu 610041, China; 3 National Key Laboratory of Science and Technology on Vacuum Electronics, School of Electronic Science and Engineering, University of Electronic Science and Technology of China, Chengdu, China

**Keywords:** Alzheimer’s disease, chaperone-mediated autophagy, lysosome, GFAP

## Abstract

Alzheimer’s disease (AD) is a progressive brain disorder marked by abnormal protein accumulation and resulting proteotoxicity. This study examines Chaperone-Mediated Autophagy (CMA), particularly substrate translocation into lysosomes, in AD. The study observes: (1) Increased substrate translocation activity into lysosomes, vital for CMA, aligns with AD progression, highlighted by gene upregulation and more efficient substrate delivery. (2) This CMA phase strongly correlates with AD’s clinical symptoms; more proteotoxicity links to worse dementia, underscoring the need for active degradation. (3) Proteins like GFAP and LAMP2A, when upregulated, almost certainly indicate AD risk, marking this process as a significant AD biomarker. Based on these observations, this study proposes the following hypothesis: As AD progresses, the aggregation of pathogenic proteins increases, the process of substrate entry into lysosomes via CMA becomes active. The genes associated with this process exhibit heightened sensitivity to AD. This conclusion stems from an analysis of over 10,000 genes and 363 patients using two AI methodologies. These methodologies were instrumental in identifying genes highly sensitive to AD and in mapping the molecular networks that respond to the disease, thereby highlighting the significance of this critical phase of CMA.

## INTRODUCTION

Alzheimer’s disease (AD), the most common cause of dementia, is one of the diseases that cause disability or premature death of the elderly in the world [1–3]. By 2021, more than 50 million people will have dementia, and AD is believed to account for 60–80% of the cases of dementia [4, 5]. The cognitive impairment and lifestyle change of AD patients have not only caused serious damage to countless families, but also posed a huge challenge to the social health system.

AD is a neurodegenerative disease that can be caused by multiple pathways. The pathways associated with AD include autophagy, inflammatory and immune responses, and lipid metabolism, among others [6–8]. Recently, some new factors that cause effects on AD have been presented, such as the coherent effect on AD between methylation and energy metabolism [9], and the miRNA effect on AD [10, 11]. These studies indicate that Alzheimer’s disease is not caused by a singular pathogenic mechanism.

Since AD is a multifactorial disease, it is a question which factor is significantly sensitive to AD.

To answer the above question, in this paper, machine learning is used to filter out genes sensitive to AD, and the Chaperone-mediated autophagy (CMA) is identified as a factor sensitive to AD. So, CMA is introduced as below.

The most prominent pathology of AD is the deposition of abnormal proteins in the brain. The aggregation of abnormal proteins leads to proteotoxicity and neuronal dysfunction. CMA, one of the three types of autophagy, actively promotes the clearance of abnormal proteins and provides effective neuroprotection [12]. In CMA, heat shock cognate 71 kDa protein (HSC70) chaperones bind to damaged or defective proteins containing the pentapeptide KFERQ-like sequences and transport them to the lysosomes for degradation via lysosome-associated membrane protein 2A (LAMP2A) [13, 14]. The main target of CMA regulation appears to be LAMP2A [15]. The intermediate filament protein glial fibrillary acidic protein (GFAP) and elongation factor 1α (EF1α, mainly encoded by EEF1A1) have been shown to be components of the lysosomal membrane that regulate LAMP2A dynamics [16]. After LAMP2A forms a multimeric complex with HSC70, the substrate needs to be unfolded and transported into the lysosome. Unphosphorylated GFAP binds to LAMP2A and stabilizes the LAMP2A multimeric complex, thereby facilitating substrate transport in CMA, while phosphorylated GFAP binds to EF1α at the lysosomal membrane [14, 16]. In the presence of GTP, EF1α is released from phosphorylated GFAP on the lysosomal membrane, allowing phosphorylated GFAP to self-assemble with GFAP molecules released from LAMP-2A [16].

CMA is associated with the accumulation of toxic proteins [17–20]. However, its relationship with AD is not well understood.

The motivation of this paper is to explore the relationship between CMA and AD. To explore the relationship, an advanced tool is necessary, different tool may lead to different discoveries. AI tool is useful. For example, the authors’ team used ant colony algorithm to discover the coherent effect on AD between methylation and energy metabolism [9], and the team used the cross-algorithm between genetic algorithm and grey wolf optimizer to filter out some gene expression characteristics of AD [21]. The integrated application of artificial intelligence, statistics, and bioinformatics appears to be more effective. For example, the team used the integrated application, it was discovered that modified folding molecular network causes effect on AD [20], the interaction causes effect on AD between T-cell antigen receptor-related genes and MAPT [22]. Not only AI method, but also the integrated application looks more useful [9, 21–23]. In this paper, the integrated application is adopted still basing on the authors’ previous accumulated experience on AD study [9, 21–23]. And AI methods [24] are used to train out the mathematics function between gene expression levels and the probability of a patient having the risk of AD, filter out the genes sensitive to AD progression, identify the molecular network sensitive to AD progression. So, CMA is drilled out. After that, the methods of statistics and bioinformatics act on CMA to explore the relationship between CMA and AD. At last, the conclusion is deduced that CMA is sensitive to AD progression.

## RESULTS

### Drill out CMA which is sensitive to AD

#### Drill out the set S_2_ that consists of the genes sensitive to AD individually

The principle of method: Genes implicated in AD are characterized by alterations in expression levels that correlate with the progression of the disease. This investigation focuses on such genes, positing that if a gene exhibits this characteristic, its expression level (*x*) is linked to the probability (*y*) of an individual being at risk for AD. Mathematically, this relationship is defined by a function *y* = *f*(*x*), where the derivative *f*′(*x*), denotes the sensitivity to AD progression. Higher values of *f*′(*x*) indicate greater sensitivity, implying that minor fluctuations in expression level (*x*) result in substantial changes in the risk probability for AD.

The aim of this section: Select the genes with the top derivative *f*′(*x*). That is, select the top genes sensitive to AD.

Method: Given that the functional relationship *f*(*x*) delineating gene expression levels and AD risk does not explicitly manifest within gene datasets, this study employs machine learning techniques to elucidate this function. Recognizing that AD is associated with not merely a single gene but an array of over 10,000 genes, the investigation expands the model to a multivariate function *y* = *f*(*x*_1_, *x*_2_, …, *x*_m_). Consequently, the concept of a singular derivative *f*′(*x*) is refined to encompass partial derivatives $\frac{\partial f}{\partial x_{i}},\;\frac{\partial f}{\partial x_{2}},\;\ldots \;,\frac{\partial f}{\partial x_{m}},$ to accommodate the multidimensional nature of gene expression’s impact on AD risk. A novel algorithm integrating machine learning with partial derivatives is developed to identify genes with the highest sensitivity to AD progression, detailed in Method.

Input data of computation: More than 10,000 genes and 363 patients, dataset GSE15222 (The details are in Method).

Output of computation: The analysis identified the top 20% of genes exhibiting significant partial derivatives, indicative of heightened sensitivity to AD progression (Table 1). These genes have been ranked according to their partial derivative values in a sequence denoted as *S_sensitivity–sequence_* or *S*_1_, with comprehensive results available in Supplementary File 1.

**Table 1:** A part of genes sensitive to AD.

**Gene name**	**Gene full name**	**Related to CMA**	**Rank**
GFAP	Glial fibrillary acidic protein	√	1
MT1F	Metallothionein 1F		2
…	…		…
EEF1A1	Eukaryotic translation elongation factor 1 alpha 1	√	53
…	…		…
HSP90AB1	Heat shock protein 90 alpha family class B member 1	√	146

The subset comprising the top 20% of genes, characterized by substantial partial derivatives and denoted as *S_sensitivity–top_* or *S*_2_, reflects a heightened sensitivity to fluctuations in gene expression levels concerning the risk of AD. In essence, s_2_ ⸦ s_1_, where *S*_2_ encapsulates those genes whose expression alterations are most closely associated with changes in AD risk probability. Key findings from *S*_2_ are detailed in Table 1.

The genes of CMA are included in the top 20% (Table 1): Genes associated with the substrate translocation into lysosomes during CMA feature prominently in the top 20% of those identified for sensitivity to AD progression (Table 1). Specifically, Table 1 indicates that GFAP emerges as the gene most sensitive to AD, securing the highest rank. Concurrently, additional genes pivotal to the process of substrate entry into lysosomes within the CMA pathway, such as EEF1A1 and HSP90AB1, also achieve top rankings, highlighting their critical sensitivity to AD.

#### Drill out the set S_4_ that consists of the genes causing molecular network sensitive to AD

The principle of method: Molecular networks underpin biological functions, exemplified by CMA, which facilitates the degradation of substrates by delivering them to lysosomes. As AD progresses, the accumulation of abnormal proteins intensifies, prompting an increased demand for such degradation processes and thereby activating CMA. Consequently, the likelihood of an individual being at risk for AD can be inferred from the activity of CMA. From a mathematical perspective, this relationship is encapsulated by a function *f*, such that *f*(*CMAgenes*), where *CMAgenes* denotes the expression levels of all genes associated with CMA, and *y* represents the probability of AD risk attributable to the CMA network. If alterations in the expression levels of CMA genes result in significant changes in AD risk probability, it suggests that CMA is highly sensitive to the disease. This insight renders machine learning an invaluable tool for deriving the function *f*, thereby enabling the quantification of CMA’s sensitivity to AD.

The aim of this section: Drill out molecular networks sensitive to AD.

Method: For example, the molecular network facilitating substrate entry into lysosomes during CMA encompasses genes such as GFAP, LAMP2A, EEF1A1, and HSP90AB1. Utilizing machine learning, we can derive the function *y* = *f*_1_(*x*_1_, *x*_2_, *x*_3_, *x*_4_), where *x*_1_, *x*_2_, *x*_3_, *x*_4_ correspond to the expression levels of GFAP, LAMP2A, EEF1A1, and HSP90AB1, respectively, with *y* denoting the AD risk probability. By isolating GFAP and recalibrating the model, a secondary function *w* = *f*_2_(*x*_2_, *x*_3_, *x*_4_) is established. The differential Δ = *y*–*w* quantifies GFAP’s impact on AD within this network, with larger Δ values indicating a more substantial influence. Given GFAP’s involvement across various molecular networks, the mean of these differential values assesses its overall effect on AD risk. The greater the average, the more pronounced is GFAP’s contribution to AD susceptibility through its network interactions. A comprehensive methodological exposition is provided in Method and the Supplementary Materials.

Input data of computation: More than 10,000 genes and 363 patients, dataset GSE15222 (The details are in Method).

Output of computation: All genes have been ranked according to their average differential value (Δ), resulting in a sorted set designated as *S_effect–sequence_* or *S*_3_ detailed in Supplementary File 2. From this ranking, the top 20% of genes have been curated into a subset, denoted as *S_effect–top_* or *S*_4_. A selection of genes within *S*_4_ is presented in Table 2.

**Table 2:** The partial list of top genes that cause molecular network sensitive to AD progression.

**Gene name**	**Gene full name**	**Related to CMA**	**Rank**
GFAP	Glial fibrillary acidic protein	√	10
…	…		…
EEF1A1	Eukaryotic translation elongation factor 1 alpha 1	√	37
…	…		…
EEF1A2	Eukaryotic translation elongation factor 1 alpha 2	√	221
…	…	…	…
HSP90AB1	Heat shock protein 90 alpha family class B member 1	√	360

The substrate translocation into lysosomes during CMA exhibits a preferential response to AD progression: As AD advances, the accumulation of abnormal proteins intensifies, leading to increased proteotoxicity and compromised proteostasis. In response, CMA is activated to transport these abnormal proteins to the lysosome, playing a crucial role in maintaining proteostasis. This selective responsiveness of CMA to AD progression is underpinned by the observation that each gene involved in the process of substrate entry into lysosomes during CMA occupies a prominent position in Table 2. This indicates that even minor variations in the expression levels of these genes significantly impact the probability of AD risk through molecular networks. Given that all genes associated with this specific CMA process are highly ranked, it demonstrates that the network governing substrate entry into lysosomes during CMA is markedly sensitive to AD, responding preferentially as the disease progresses.

#### Filter out subset S_6_ from S_2_ ⋂ S_4_ which is related to CMA

In “Drill out the set *S*2 that consists of the genes sensitive to AD individually” section, the gene set *S*_2_ is characterized by its constituents’ heightened sensitivity to AD progression, with each gene within the set demonstrating a discernible response to the disease’s advancement. Conversely, by “drilling out the set *S*_4_ that consists of the genes causing molecular network sensitive to AD” section, the gene set *S*_4_ is introduced, which embodies a distinct trait: the expression level changes of any given gene within this set—and the molecular network encompassing it—result in a significantly pronounced effect on AD progression through the network. This suggests that the gene is crucial within its network, rendering the network itself particularly sensitive to AD progression.

By intersecting *S*_2_ and *S*_4_, a new set is defined, *S*_5_ = *S*_2_ ⋂ *S*_4_, comprising 1,575 genes. These genes simultaneously exhibit the aforementioned characteristics, implying that networks with particular sensitivity to AD progression are embedded within *S*_5_. However, the task of visually identifying these sensitive networks from the substantial dataset of 1,575 genes is beyond the scope of human analytical capabilities, given the vastness of the information presented. Consequently, traditional bioinformatics methodologies are applied to *S*_5_ in this section to navigate through and analyze the extensive data.

**Figure 1:**
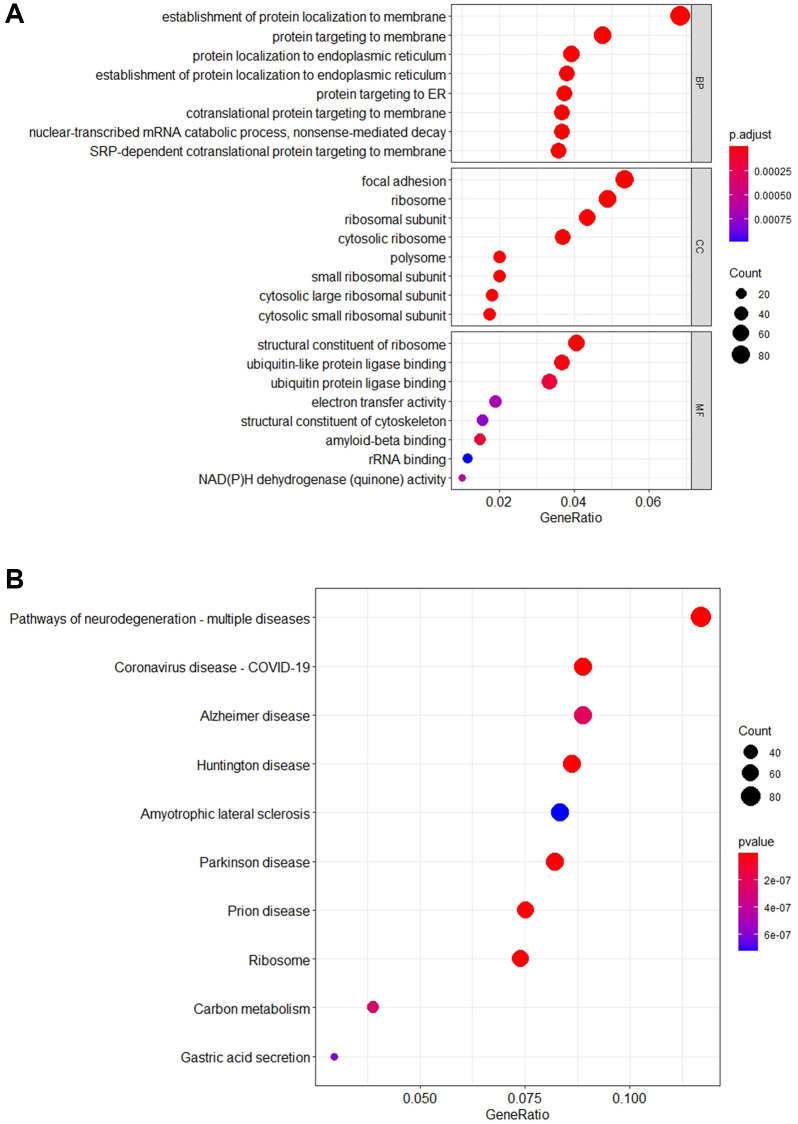
**The schematic diagram of enrichment analysis KEGG and GO.** (**A**) The bubble diagram shows the top 10 pathways that are enriched for important genes. The results show that most of the *S*_5_ genes are enriched in the “Pathways of neurodegeneration-multiple diseases” pathway. In addition, these genes are mainly involved in “Alzheimer Disease”, “Huntington Disease” and other neurodegenerative pathways. (**B**) The bubble diagram shows the top 8 GO terms that are enriched for important genes. The vertical axis corresponds to the Biological Process (BP), Cell Component (CC), and Molecular Function (MF). The results indicate that most of the *S*_5_ genes are involved in “establishment of protein localization to membrane” and “protein targeting in membrane” biological processes.

Enrichment analysis conducted on set *S*_5_ yields insights delineated in Figure 1, through both Kyoto Encyclopedia of Genes and Genomes (KEGG) and Gene Ontology (GO) analyses. KEGG analysis categorizes genes in relation to specific diseases and biochemical pathways [25, 26], while GO analysis organizes genes based on their molecular functions and biological processes [27]. According to KEGG, genes within *S*_5_ are significantly represented in pathways associated with various brain disorders, notably including “neurodegenerative-multiple disorders,” “Alzheimer’s disease,” and “Huntington’s disease” (Figure 1A). GO analysis reveals a predominant enrichment in biological processes such as “establishment of protein localization to membranes’ and “protein targeting to membranes” (Figure 1B), suggesting a crucial role in cellular functionality. Further details are available in Supplementary File 3.

These findings indicate that the genes in set *S*_5_ are implicated in Alzheimer’s disease, either individually or through specific gene networks, with a significant number participating in pathways relevant to neurodegenerative diseases. Notably, their cellular functions are chiefly enriched in processes related to protein localization and targeting to membranes, underscoring their potential roles in the pathological mechanisms underlying AD.

Given the prominent ranking of GFAP in both preceding analyses, our attention pivoted to biological networks featuring GFAP, as identified in the Gene Ontology (GO) analysis. The pertinent GO term associated with GFAP emerged as “regulation of protein catabolic process”. The gene set encapsulated by this term, designated as *S_catabolic_* or *S*_6_, comprises 51 genes distinguished by their acute sensitivity to AD. These genes are posited to exert influence on AD progression through their involvement in biological functional networks. Notably, *S*_6_ includes genes related to CMA, and forthcoming analyses will assess the significance of these CMA-related genes within *S*_6_. Detailed information on the genes constituting *S*_6_ is provided in Supplementary File 4.

#### CMA is induced from set S_6_

**Figure 2:**
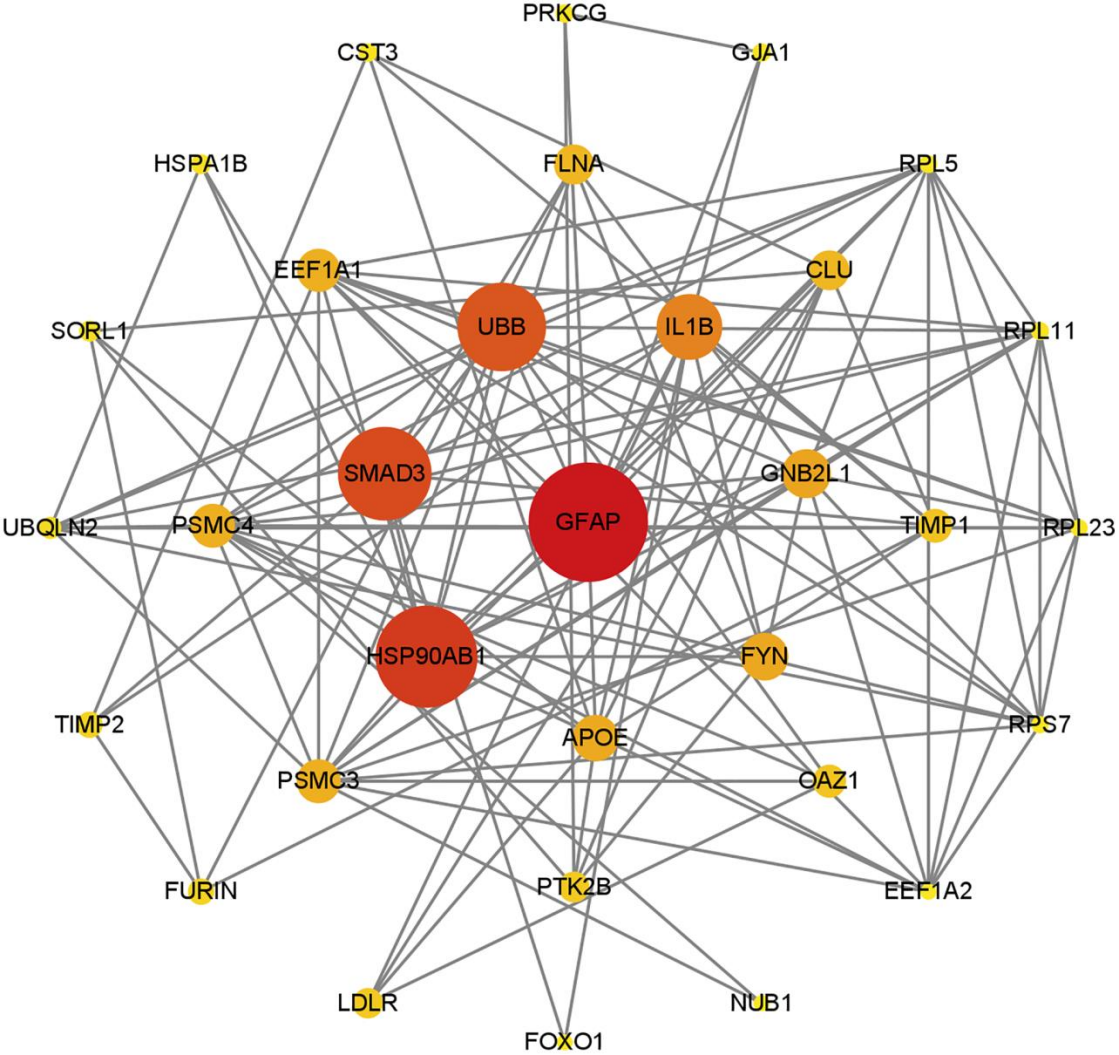
**PPI network map of genes contained in *S*_6_, was constructed based on STRING database and visualized by Cytoscape.** The ranking was performed after filtering by the betweenness centrality algorithm, with nodes closer to the center or colored closer to red indicating higher scores. The results show that GFAP still dominates in this network, and other genes associated with CMA are close to the center of the network.

The STRING database facilitates the exploration of potential associations among genes based on functional interactions. Utilizing this resource, the gene set *S*_6_ underwent analysis for Protein-Protein Interactions (PPI) to construct a gene network. Subsequent evaluation of network topology was performed through the Betweenness Centrality algorithm, calculating the centrality of each node. The findings are visually represented in Figure 2, where a gene’s proximity to the center signifies its pivotal role within the network. Notably, GFAP emerged as the most central gene, registering the highest centrality score (172.77), followed by HSP90AB1 (143.75), SMAD3 (129.80), and UBB (121.34). Detailed centrality scores for the remaining genes are accessible in Supplementary File 5.

This network revealed that the main functions of the gene that plays a dominant role in collection *S*_6_ were all related to chaperone-mediated autophagy. Genes close to the center such as GFAP, HSP90AB1 and EEF1A1 are involved in chaperone-mediated autophagy. Thus, the results suggest that CMA plays an important role in AD.

The analysis of the network underscores that the primary functions of genes central to the set *S*_6_ are intrinsically linked to the process of substrate translocation into lysosomes during CMA. Key genes situated near the network’s core, such as GFAP, HSP90AB1, and EEF1A1, play pivotal roles in this specific autophagy process, highlighting CMA’s significant contribution to AD pathology.

Figure 2 illustrates the critical involvement of several CMA-associated genes, including GFAP, HSP90AB1, and EEF1A1, in AD, suggesting a nuanced understanding of their impact. To refine the assessment of their effects on AD, the analysis incorporates LAMP2, the gene encoding the lysosome-associated membrane protein 2A (LAMP2A), leading to an updated gene set *S*_7_.

Figure 2 shows that most of the genes in CMA, such as GFAP, HSP90AB1 and EEF1A1, play important roles in AD. In order to more precisely assess the effect on AD, LAMP2 is considered, which encodes the protein LAMP2A. Then update gene set *S*_6_ and get set *S*_7_.


$${\text{S}}_{7}={\text{S}}_{CMA}=\text{\{}GFAP\text{,}\;HSP\text{90}AB\text{1,}\;EEF\text{1}A\text{1,}\;LAMP\text{2\}}$$

The genes encompassed by set *S*_7_ encode proteins critical to the process of substrate translocation into lysosomes, a key aspect of CMA, thereby aligning *S*_7_ closely with this specific phase of CMA. Consequently, *S*_7_ is designated as representative of this crucial autophagic pathway, henceforth denoted as *S_CMA_*. The characteristics and significance of are outlined in Table 3, which clarifies the relationship between this targeted aspect of CMA and AD, emphasizing the susceptibility of this autophagic route to AD’s progression.

**Table 3:** The characterization of set *S_CMA_*.

**Characterization of *S_CMA_***	**Description**
Sensitivity to AD	The genes of *S_CMA_* are sensitive to AD individually excluding LAMP2 (“Drill out the set *S*_2_ that consists of the genes sensitive to AD individually” section)
Causing molecular network sensitive to AD	CMA preferentially responds to AD progression (“Drill out the set *S*_4_ that consists of the genes causing molecular network sensitive to AD” section)
Pathway enrichment (KEGG)	Primarily involved in neurodegenerative disease pathways, including AD (“Filter out subset *S*_6_ from *S*_2_ ⋂ *S*_4_ which is related to CMA” section)
Function enrichment (GO)	The GO term "regulation of protein catabolic process" contains GFAP with the highest confidence level (“Filter out subset *S*_6_ from *S*_2_ ⋂ *S*_4_ which is related to CMA” section)
PPI analysis	These genes were located at the center of the network, suggesting an important role in their biological function (“CMA is induced from set *S*_6_” section)

### The analysis of CMA characterization

#### Differential expression analysis of CMA

**Figure 3:**
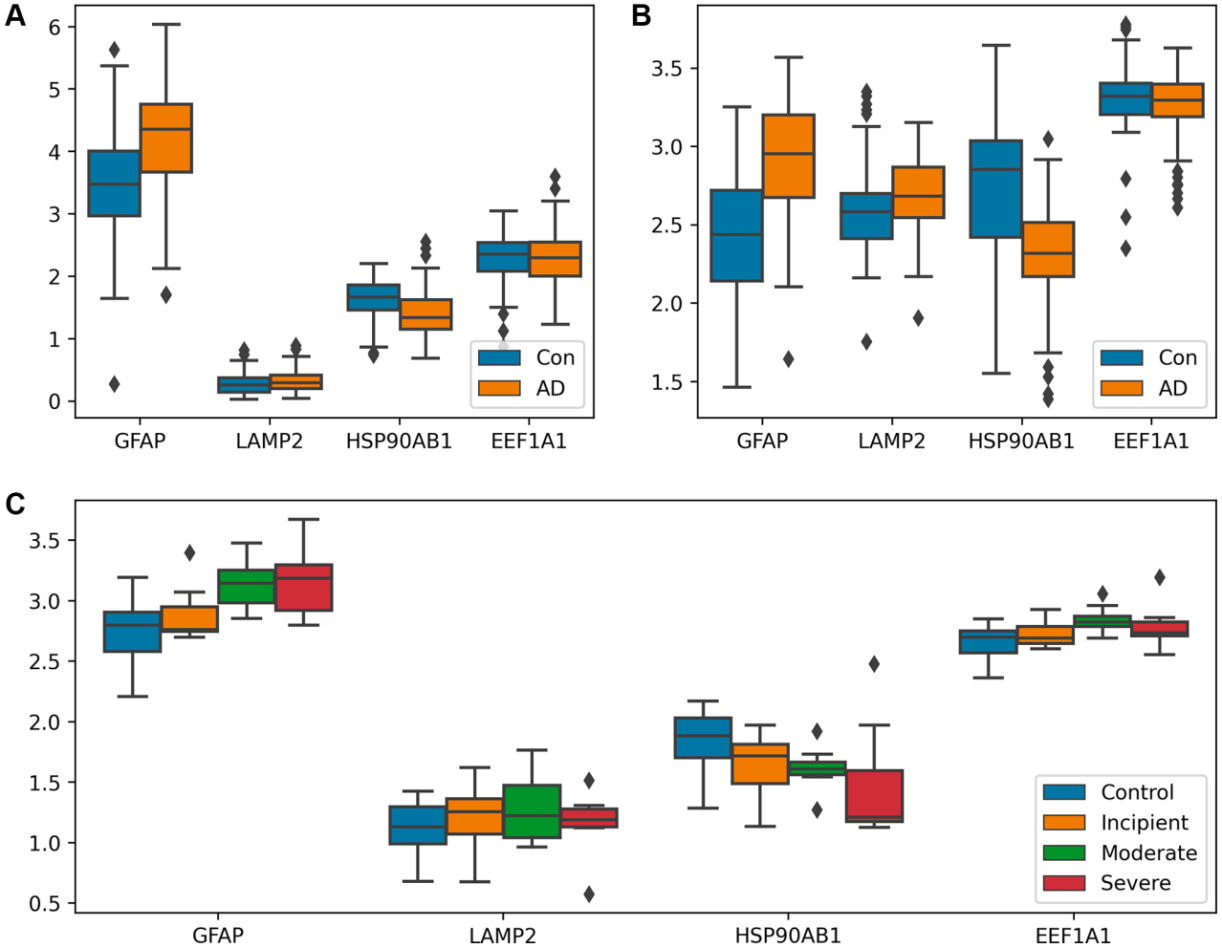
**Analysis of differential expression between AD patients and controls.** (**A**, **B**) Box plots of CMA-related genes differentially expressed in GSE15222 and GSE5281, distinguishing the AD group from the control group. Both GFAP and LAMP2 showed a trend of upregulation in (**A**) and (**B**), and HSP90AB1 shows a different trend. (**C**) Box-plot of CMA-related genes differentially expressed in GSE1297, shown according to control, incipient dementia, moderate dementia, and severe dementia. GFAP expression gradually increased with increasing dementia in (**C**). Additionally, the *T*-test was utilized to verify the significance of gene expression, with detailed results available in Supplementary File 6.

Following the identification of the gene network *S*_7_, which is associated with the process of substrate translocation into lysosomes within CMA, we proceeded to examine the expression profiles of genes within this network. Differential expression analysis was carried out between AD cohorts and control groups, utilizing three separate datasets. The results of this analysis are visually detailed in Figure 3, providing insight into the expression patterns of these genes in the context of AD.

GFAP has been documented as a regulator of LAMP-2A assembly/depolymerization through a GTP-dependent mechanism, consequently influencing the pace of CMA [16, 28]. As a result, the observed upregulation of GFAP during the Alzheimer’s disease phase implies a positive regulation of CMA facilitated by GFAP. The rate of CMA is also linked to the abundance of LAMP-2A within the lysosomal membrane [16, 28]. The quantity of LAMP-2A, in turn, is subject to regulation through transcriptional upregulation [28, 29]. The result showed a significant expression of LAMP2 during AD, implying the activation of CMA at the outset of AD, *T*-test was used to verify the significance of the genes. Additionally, HSP90AB1, a member of the HSP90 chaperone protein family, helps stabilize protein folding [30]. HSP90 is believed to have the potential to unfold substrates that have already folded in complexes on the lysosomal membrane [31, 32]. Thus, its downregulation assists in unfolding substrates, making it easier for them to enter lysosomes. Collectively, these results suggest that the process of substrate translocation into lysosomes, a critical component of CMA, is upregulated in AD to augment the degradation of abnormal substrates.

#### Correlation analysis of CMA

Given the synergistic interactions between genes involved in the process of substrate translocation to the lysosome, it becomes important to study their interrelationships. Accordingly, this section is dedicated to the calculation of correlation coefficient matrices.

**Figure 4:**
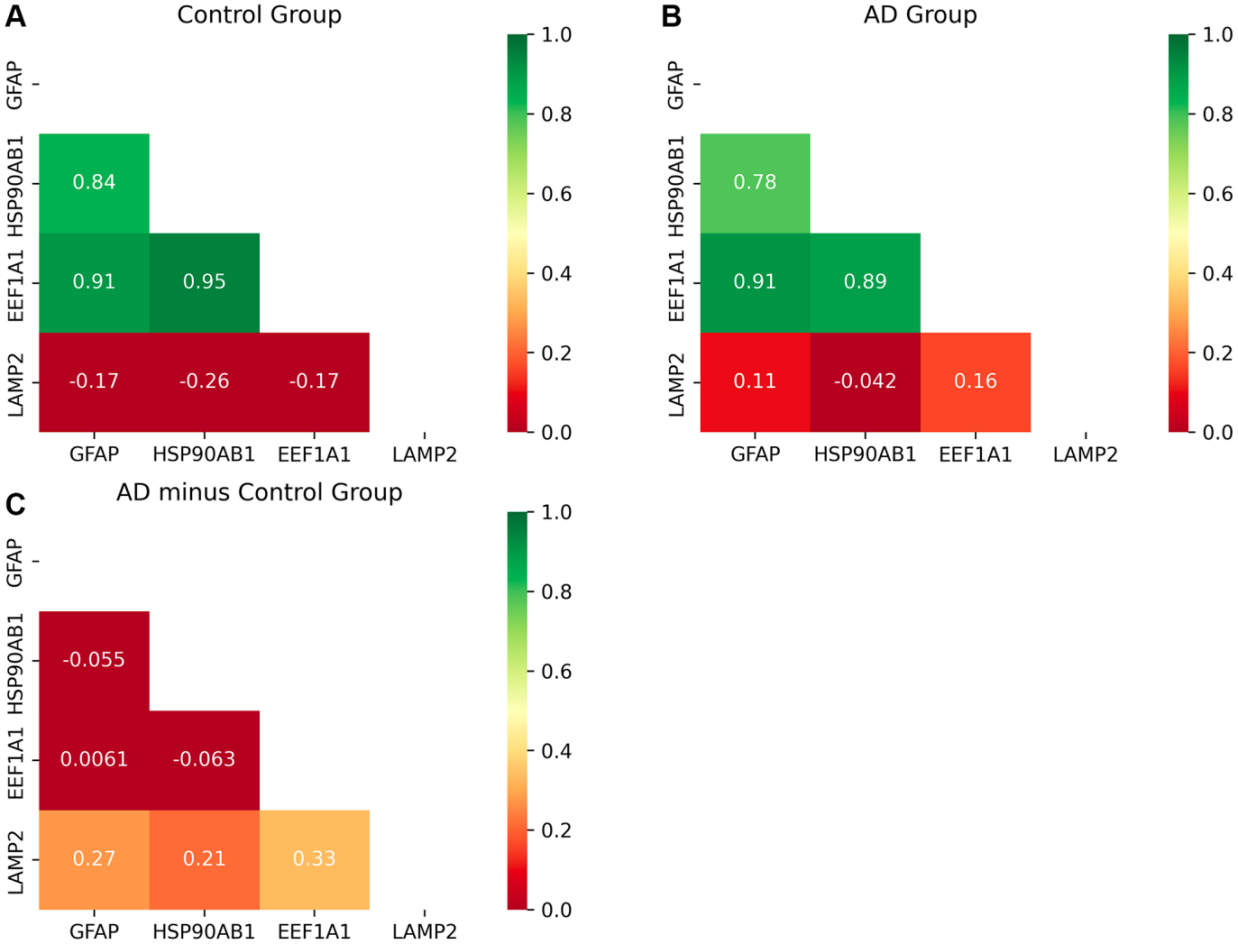
**Heat map of correlation matrices of the proteins of CMA.** (**A**, **B**) The correlation coefficient matrix among the proteins of CMA. (**C**) The difference matrix. Figure 4C shows that the degree of correlation between LAMP2 and the other three genes become stronger significantly as AD progresses. And the other three genes keep strong correlations among them. The inhibitory protein HSP90 unfolds substrates ready to be delivered to the LAMP2A complex for degradation, so the correlation between them becomes stronger. After unfolding, LAMP2A works with GFAP to deliver substrates to lysosome together, so the correlation becomes stronger. After finishing the delivery, the protein encoded by EEF1A1 dissociates GFAP to restore the LAMP2A complex in CMA, so the correlation becomes stronger. Thus, CMA becomes active with AD progression. The more detailed explanation of the molecular mechanism is described in Conclusion and Figure 6.

The input data for this analysis are derived from the GSE15222 dataset. The outputs are matrices representing the correlation coefficients, with one derived from control group data (Figure 4A) and the other from AD patient data (Figure 4B). The difference matrix between the control group and the AD group indicates changes in correlation (Figure 4C).

Upon comparison of the control and AD groups, two notable observations emerge:

1. The robust correlation among GFAP, HSP90AB1, and EEF1A1 is maintained, indicative of their concerted function within the process of substrate translocation into lysosomes, a pivotal aspect of CMA. Specifically, HSP90AB1 is implicated in the initiation of this substrate delivery process. GFAP plays a critical role in the delivery action itself, crucial for stabilizing the CMA complex. The protein encoded by EEF1A1 facilitates the completion of substrate delivery. These operational dynamics of CMA, particularly in relation to substrate translocation into lysosomes, are discussed in detail in Discussion and Conclusion.
  Consequently, the observed strong correlation among these genes underscores the heightened activity of this specific autophagic pathway during the progression of AD, suggesting its vital role in responding to the disease’s advancement.
2. As AD progresses, LAMP2 exhibits an increasing correlation with other genes involved in the process of substrate translocation into lysosomes, a critical function of CMA. In the substrate delivery phase, GFAP and LAMP2A collaborate to form a translocation complex essential for substrate movement into lysosomes. Here, LAMP2A plays a direct role in the delivery, while GFAP contributes to the stability of this process, as detailed in Discussion and Conclusion. Therefore, the observed enhancement in gene correlation indicates that the substrate translocation aspect of CMA intensifies in activity during AD progression, facilitating increased substrate degradation.

#### The relationship between CMA and dementia degree

In this section, linear regression analysis is employed to investigate the relationship between the process of substrate translocation into lysosomes during CMA and key clinical or anatomical indicators of AD progression.

The clinical indicator under consideration is the Mini-Mental State Examination (MMSE), with lower scores on this assessment indicating more severe dementia. The MMSE score serves as a reflection of AD progression. The anatomical indicator examined is the presence of Neurofibrillary Tangles (NFTs), where a higher count is associated with an increased degree of dementia. NFTs signify the accumulation of proteotoxicity, further correlating with the disease’s advancement.

**Figure 5:**
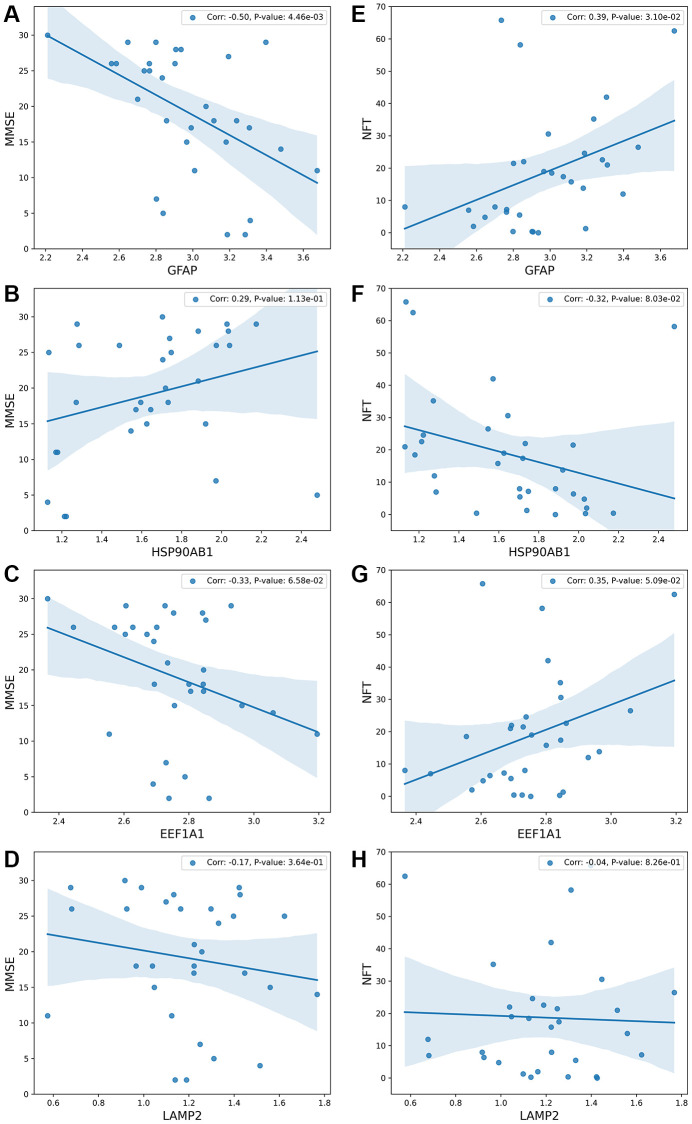
**The relationship between the process of substrate translocation into lysosomes during CMA and dementia degree.** (**A**–**D**) detail the correlation between gene expression levels and the Mini-Mental State Examination (MMSE) scores, which serve as a clinical measure of dementia severity, with lower MMSE scores indicating more severe dementia. The vertical axis denotes MMSE scores, while the horizontal axis captures gene expression levels. (**E**–**H**) explore the link between gene expression levels and the count of Neurofibrillary Tangles (NFTs), markers of neurodegeneration. The underlying molecular mechanism across these subfigures highlights that CMA’s role in degrading substrates—generally abnormal proteins—is triggered by substrate accumulation. Excessive accumulation of such proteins results in proteotoxicity, correlating with increased dementia severity. Subfigure B demonstrates that lower expression of HSP90AB1 aligns with reduced MMSE scores and heightened dementia severity. HSP90AB1 functions as an inhibitory protein; its reduced expression facilitates the unfolding of abnormal proteins, easing their entry into the LAMP2A complex and thus activating CMA. Consequently, lower levels of HSP90AB1 indicate enhanced CMA activity. Subfigures A and D show that higher expressions of GFAP or LAMP2 correlate with lower MMSE scores and increased dementia severity. The GFAP-LAMP2A complex is essential for delivering substrates to the lysosome, and its activation is prompted by the overaccumulation of abnormal proteins. The presence of more severe dementia suggests greater protein accumulation, leading to increased activity of the GFAP-LAMP2A complex and elevated expression of both GFAP and LAMP2. Upon completion of substrate delivery, the protein encoded by EEF1A1 disassociates GFAP, resetting the LAMP2A complex to its initial state, as depicted in subfigure C. Higher levels of EEF1A1, indicating lower MMSE scores and greater dementia, underscore its role in concluding the delivery process and reinstating CMA’s baseline functionality. Overall, the sensitivity of the process of substrate translocation into lysosomes during CMA to AD progression mirrors the degree of dementia, offering a reflective measure of dementia severity. This comprehensive analysis is further detailed in Conclusion and illustrated in Figure 6.

Input data are from dataset GSE1297, and the output is shown in Figure 5. Figure 5A–5D show the correlation between four genes and MMSE. Figure 5E–5H show the correlation between four genes and NFT.

**Figure 6:**
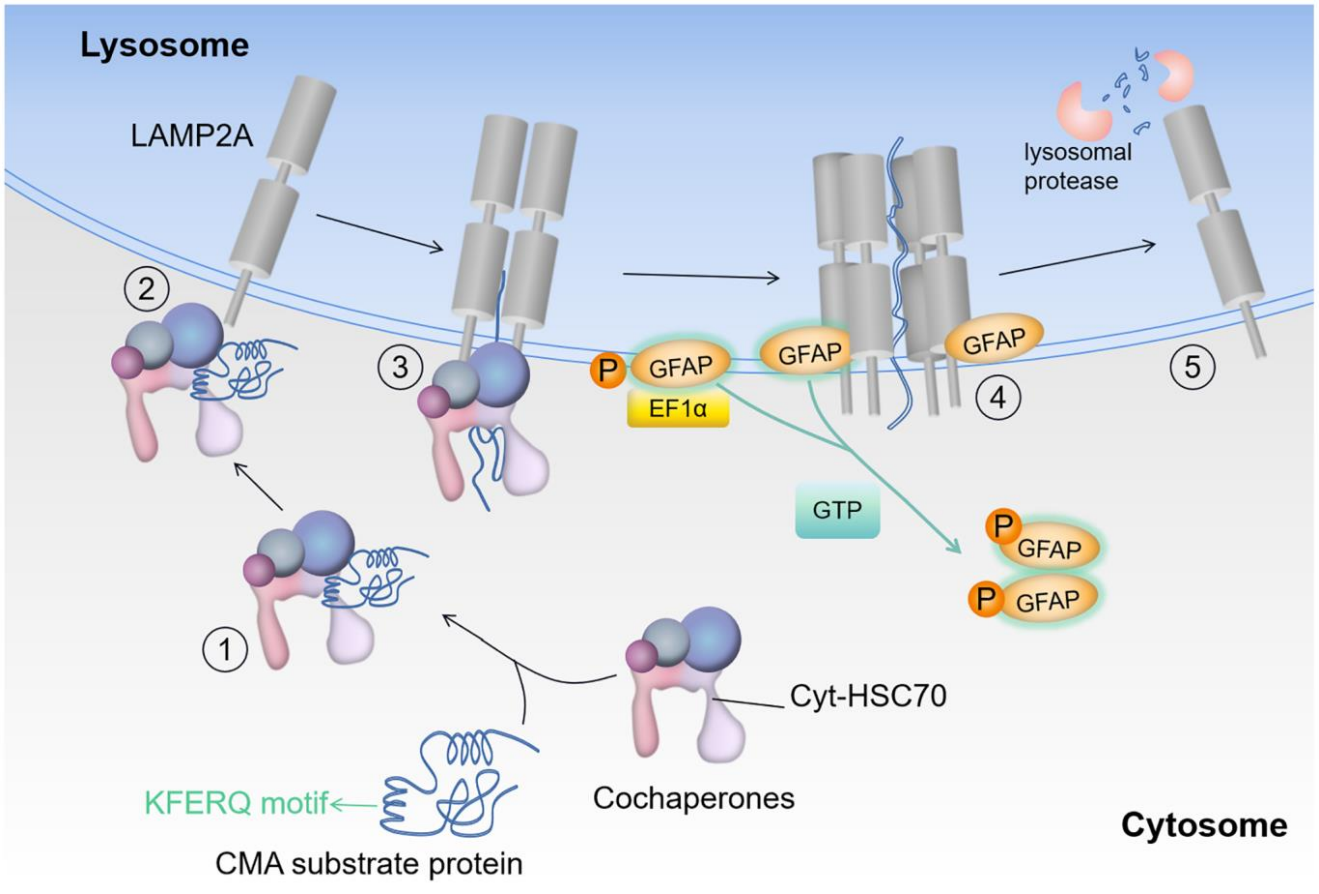
**Substrate entry into the lysosome.** Protein degradation by CMA: HSC70 recognizes the KFERQ-like motif in the substrate (step 1); the substrate-chaperone complex binds to LAMP2A (step 2); the chaperone complex expands the substrate to form the CMA translocation complex (step 3); substrate translocation is mediated by other proteins in the lysosome, when GFAP acts as a reinforcer of the complex (step 4); lysosomal protease degrades the substrate and LAMP2A dissociates from the translocation complex (step 5). Where EF1α denotes elongation factor 1-α (core subunit is EEF1A1), GFAP denotes glial fibrillary acidic protein, and HSC70 denotes heat shock cognate 71 kDa protein (also known as HSPA8).

Subfigure A illustrates that with an increasing degree of dementia, the MMSE scores decrease while the expression of GFAP rises. Subfigure E demonstrates that excessive proteotoxicity accumulation results in both elevated NFT counts and increased GFAP expression. To interpret these observations, the paper proposes a rationale: as AD progresses, abnormal proteins accumulate, activating the process of substrate translocation into lysosomes within CMA for clearance. GFAP, playing a pivotal role in this process, sees its upregulation as critical for mitigating proteotoxicity accumulation. This mechanism is elaborated upon in Discussion and Conclusion, and illustrated in Figure 6.

Subfigure B correlates higher degrees of dementia (reflected by lower MMSE scores) with reduced expression of HSP90AB1, while Subfigure F connects proteotoxicity accumulation with both increased NFT and decreased HSP90AB1 levels. HSP90AB1 acts as an inhibitory protein, and its downregulation facilitates the unfolding of abnormal proteins, easing their entry into the lysosomal degradation pathway.

Subfigure C correlates increasing severity of dementia (reflected in lower MMSE scores) with an upsurge in EEF1A1 expression. Similarly, Subfigure G associates heightened proteotoxicity (evidenced by elevated NFT counts) with increased EEF1A1 levels. The EEF1A1 protein is instrumental in dissociating GFAP, thereby facilitating the reconstitution of the LAMP2A complex, crucial for completing and resetting the process of substrate translocation into lysosomes during CMA. Consequently, an increase in EEF1A1 expression indicates an activation of this specific phase of CMA.

Subfigure D shows that greater dementia severity is associated with lower MMSE scores and higher LAMP2 expression. According to Discussion and Conclusion, the GFAP and LAMP2A complex forms the core unit of this autophagic pathway, crucial for the translocation of substrates into lysosomes for degradation. Elevated expressions of GFAP and LAMP2A affirm the operational status of this pathway in response to AD progression.

In summary, the process of substrate translocation into lysosomes within CMA exhibits sensitivity to the progression of AD, thereby exerting a notable impact on clinical indicators.

#### CMA is a biomarker of AD

**Figure 7:**
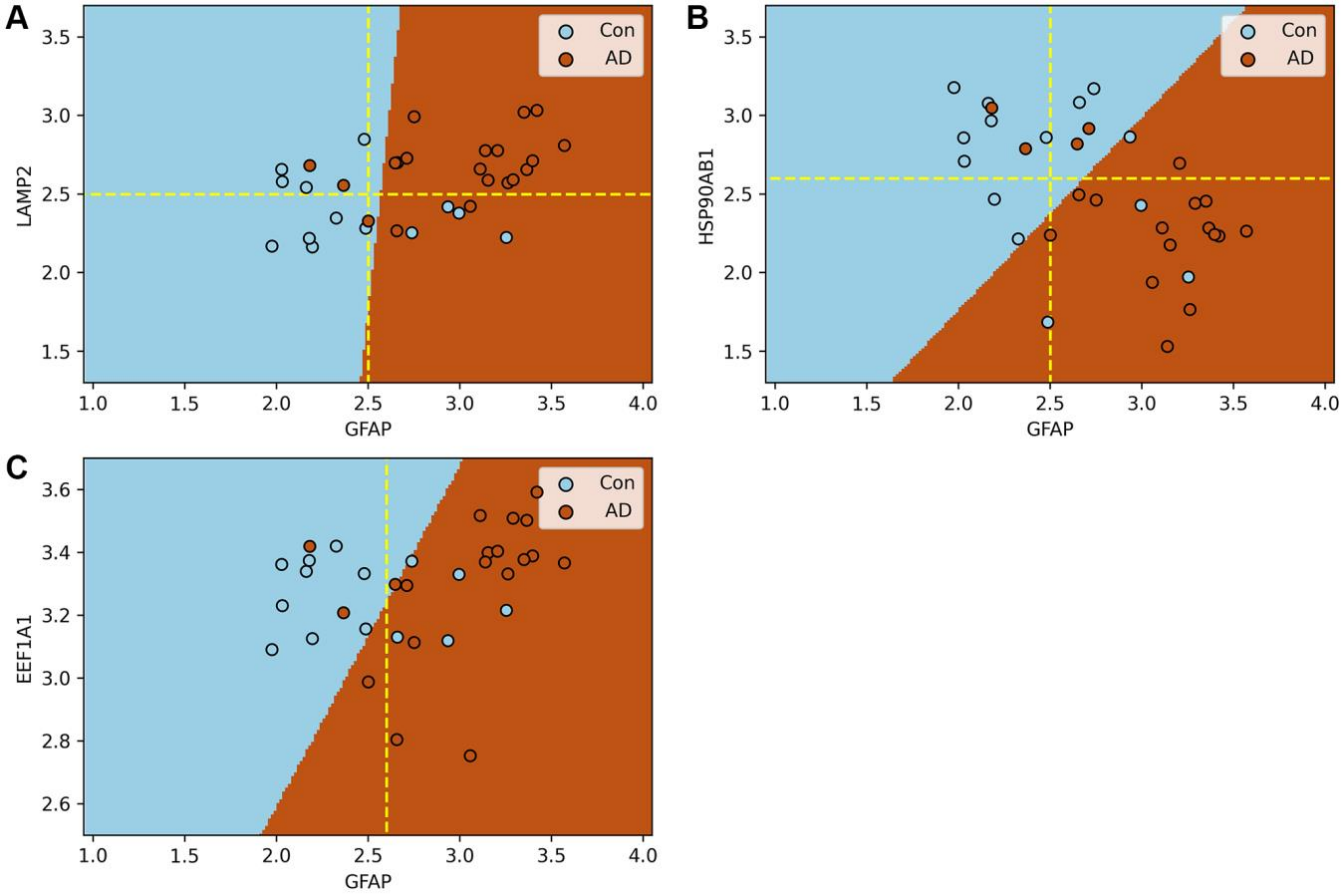
**Support vector machine models of the process of substrate translocation into lysosomes during CMA.** (**A**–**C**) plot the expression of GFAP against that of other key CMA proteins. Here, blue and brown markers represent control and AD groups, respectively, with dashed lines indicating critical expression thresholds. Subfigure A demonstrates that when both LAMP2A and GFAP expressions surpass their thresholds, the risk of AD nears certainty. Subfigure B shows a heightened AD risk when HSP90AB1 falls below its threshold, while GFAP’s expression is above its own. Collectively, these models confirm the sensitivity of the process of substrate translocation into lysosomes during CMA to the progression of AD, highlighting its potential as a biomarker. The molecular rationale underlying these observations involves CMA’s activation in response to the excessive accumulation of abnormal proteins due to AD progression, necessitating a three-step process for substrate degradation. Initially, HSP90AB1 facilitates substrate unfolding to prepare for lysosomal delivery. Subsequently, LAMP2A and GFAP collaborate to form a translocation complex, efficiently directing substrates to the lysosome. Finally, EEF1A1 disengages GFAP from the complex, resetting LAMP2A for subsequent cycles. These stages correspond to the findings depicted in Subfigures B, A, and C, respectively. Subfigure B underscores the initial response of the substrate translocation process into lysosomes within CMA to proteotoxicity accumulation, a critical factor in AD risk assessment. Subfigure A showcases the delivery phase, where the combined actions of LAMP2A and GFAP, manifested through their increased expression levels, significantly boost the process’s capacity to eliminate proteotoxic accumulations. This stage indicates the proactive engagement of this specific CMA phase in substrate degradation. Thus, the integrated function of this lysosomal entry process, rather than the action of individual proteins, stands out as a prominent biomarker for AD. A more comprehensive explanation of this process and its implications for AD diagnosis is provided in Conclusion and illustrated in Figure 6.

In this section, the impact of the process of substrate translocation into lysosomes within CMA on AD is evaluated using a Support Vector Machine model (SVM). The dataset GSE5281 serves as the input, with the findings depicted in Figure 7. In addition, the results of ten-fold cross validation can be found in Supplementary Figures 1–3.

Figure 7A illustrates that when both GFAP and LAMP2A—proteins integral to the lysosomal substrate translocation process of CMA—are upregulated beyond their respective thresholds, the likelihood of AD risk in a patient approach nearly 100%.

Figure 7B reveals that a significantly high probability of AD risk is observed when HSP90AB1 falls below a critical level, while GFAP exceeds its threshold.

Figure 7C indicates that within the control group, EEF1A1 expression is confined to a specific range. When the expression extends beyond this range, coupled with GFAP exceeding its threshold, the samples are classified as AD.

It is crucial to acknowledge that relying on a single protein as a biomarker has its limitations. For instance, as shown in Figure 7C, despite GFAP exceeding its threshold (indicated by the dashed line), five samples remain within the control category. Similar observations are noted in the other subfigures.

The SVM model elucidates that GFAP, in conjunction with LAMP2 and HSP90AB1, exhibits a synergistic interaction affecting AD. If they are both input into the model, the accuracy of the model’s prediction can reach 85%. Collectively, the ensemble of proteins involved in the lysosomal substrate translocation phase of CMA acts as a robust biomarker for AD, whereas individual proteins demonstrate limited biomarker efficacy.

## DISCUSSION

AD is a brain disorder that gets worse over time. It’s characterized by changes in the brain that lead to deposits of abnormal proteins. The aggregation of abnormal proteins leads to proteotoxicity and neuronal dysfunction. CMA is a lysosomal pathway of proteolysis that is responsible for the degradation of cytosolic proteins, and it contributes to cellular quality control through the removal of damaged or malfunctioning proteins. On the one hand, the over-accumulation of abnormal proteins accelerates the progression of AD. On the other hand, CMA participates in degradation to clear up the over-accumulation and slows down the progression. The game between the two actions of accumulation and clearance affects the progression of AD.

The process of substrate translocation into lysosomes within CMA constitutes a molecular network involving key proteins such as GFAP, LAMP2A, HSP90AB1, and EEF1A1. These proteins, encoded by their respective genes, collaborate integrally to facilitate the lysosomal degradation of substrates. Functioning collectively, this network features the chaperone protein HSP90, encoded by HSP90AB1, which plays a crucial role in modulating substrate unfolding [30–32]. Concurrently, the GFAP and LAMP2A complex is essential for the actual translocation of substrates into the lysosome, whereas the protein produced by EEF1A1 concludes this delivery phase [14, 16]. Together, these components underscore the orchestrated operation of the CMA pathway, particularly its critical phase of moving substrates into lysosomes for degradation.

On one side, the excessive accumulation of substrates results in proteotoxicity, contributing to the accelerated progression of AD and elevating the likelihood of AD risk in individuals. On the flip side, the process of substrate translocation into lysosomes, a key facet of CMA, responds to this over-accumulation by facilitating the degradation of these substrates. Consequently, it is plausible that the genes associated with this phase of CMA exhibit specific expression patterns in response to proteotoxicity accumulation, thereby mirroring the individual’s risk of AD.

This paper aims to dissect the intricate relationship between the lysosomal entry process of CMA and AD progression. More precisely, it seeks to investigate how the gene expression profiles pertinent to this particular phase of CMA correlate with the probability of AD risk, providing insights into the molecular underpinnings of the disease’s development.

Driven by the above motivation, two methods are proposed in this paper, and they aim at the two functions of computation. One is to estimate the patient’s probability of AD risk, the other is to identify the molecular network sensitive to the change of probability.

To reach the first aim of computation, the improved machine learning is designed (“Drill out the set *S*_2_ that consists of the genes sensitive to AD individually” section, “The method to identify the genes sensitive to AD” section). The method is abstracted into the following mathematics model. The relationship between gene expression levels and the probability of AD risk is defined as a mathematic function *y* = *f*(*x*_1_, *x*_2_, …, *x_m_*), where, *x*_1_, *x*_2_, …, *x_m_* denotes the expression levels of *m* genes respectively and these data are sampled from a patient or sample, *y* denotes the probability that the patient has a risk of AD. Function *y* = *f*(*x*_1_, *x*_2_, …, *x_m_*) represents that, for a given patient, his probability of having AD risk can be assessed from the expression levels of *m* genes. And partial derivative $\frac{\partial f}{\partial x_{1}},\;\frac{\partial f}{\partial x_{2}},\;\ldots \;,\frac{\partial f}{\partial x_{m}}$ is used to measure the degree of sensitivity to AD of every gene respectively. For example, if the value $\frac{\partial f}{\partial x_{1}}$ is big, the little change of the expression level of gene No. 1 leads to a significant change of the probability of AD risk. That is, the gene No. 1 is sensitive to AD. The function *y* = *f*(*x*_1_, *x*_2_, …, *x_m_*) is trained out using the proposed AI method in “The method to identify the genes sensitive to AD” section. Then, the genes highly sensitive to AD are identified by the function *f*, and contained in set *S*_2_.

To reach the second aim, the other AI method is proposed (“Drill out the set *S*_4_ that consists of the genes causing molecular network sensitive to AD” section, “The method to identify the genes sensitive to AD through molecular network” section). And its idea is illustrated using the process of substrate translocation into lysosomes during CMA as an example. The probability of AD risk caused by this process can be calculated by the above function *y* = *f*(*x*_1_, *x*_2_, *x*_3_, *x*_4_,), where *x*_1_, *x*_2_, *x*_3_, *x*_4_ denotes the expression level of the four genes in this process. The probability is labeled as *f*[*CMA*], which measures the effect of CMA on AD risk. That is, for a given patient, his probability of AD risk is reflected by the efficiency of CMA delivering substrates to lysosome, and the probability is assessed by *f*[*CMA*]. Delete GFAP from the set of CMA, and label the updated set as *CMA –* {*GFAP*}. AI Training method acts on the updated set and gets a new function *g*, then probability is calculated, and is labeled as *g*[*CMA –* {*GFAP*}]. Let *Δ*(*GFAP*) *= f*[*CMA*] – *g*[*CMA –* {*GFAP*}], where *Δ*(*GFAP*) is the difference of probability, which measures the contribution of GFAP to network CMA. The bigger *Δ*(*GFAP*), the stronger the ability of GFAP that regulate the probability of AD risk caused by network CMA. GFAP causes the effect on AD through molecular networks in general, not through GFAP individually. And GFAP participates in many networks to cause an effect on AD synthetically. Then, every network generates a value, the average of all values appears, and the average is labeled as, $\bar{\text{Δ}(\text{GFAP})}.$ The average looks more reasonable to assess the effect on AD caused by GFAP through networks. For any protein, its effect on AD through molecular networks can be assessed, such as $\bar{\text{Δ}(\text{LAMP2})},$  $\bar{\text{Δ}(\text{HSP90AB1})},$ and $\bar{\text{Δ}(\text{EEF1A1})}.$ In “Drill out the set *S*_4_ that consists of the genes causing molecular network sensitive to AD” section, for more than 10,000 genes, the average score of each one is calculated, and the genes with a high score are collected in set *S*_4_.

Then every gene included in the intersection *S*_2_ ∩ *S*_4_ holds two features. One feature is that, the gene is individually sensitive to AD. And the other feature is that, the gene is sensitive to AD through molecular network. Because the process of substrate translocation into lysosomes during CMA is a subset of *S*_2_ ∩ *S*_4_ and every gene in this process holds a high score, this process is sensitive to AD significantly. In addition, traditional bioinformatics methods act on *S*_2_ ∩ *S*_4_ to confirm that this process is related to AD in this paper.

Using the above two AI methods, the process of substrate translocation into lysosomes during CMA is drilled out. And its four characteristics are discovered and listed as below.

1. As AD progresses, the process of substrate translocation into lysosomes, a key phase of CMA, exhibits increased activity. This enhanced activity is underscored by two principal characteristics: the upregulation of proteins that facilitate this process or the downregulation of inhibitory proteins, as illustrated in Figure 3, and the strengthening of correlations among the genes involved in this specific phase of CMA as AD advances, demonstrated in Figure 4. A higher degree of correlation among these genes signifies a more robustly active process of substrate translocation into lysosomes, leading to more efficient degradation of abnormal proteins implicated in AD.
2. The process of substrate translocation into lysosomes during CMA preferentially responds to AD progression (“Drill out the set *S*_4_ that consists of the genes causing molecular network sensitive to AD” section).
3. The process of substrate translocation into lysosomes within CMA exhibits a correlation with clinical indicators of AD, as depicted in Figure 5. With an increase in the severity of dementia, there is a corresponding intensification in the accumulation of proteotoxicity. This scenario prompts a heightened activity in this specific phase of CMA, aimed at enhancing the degradation of abnormal proteins associated with the progression of AD.
4. The synergistic interaction of proteins involved in the process of substrate translocation into lysosomes during CMA functions as an indicator of AD, as evidenced in Figure 7. Specifically, when proteins such as GFAP and LAMP2A, which are key to this phase of CMA, are concurrently upregulated beyond their respective thresholds, there exists a near-certain risk of AD for the patient, as illustrated in Figure 7A.

In sum, the process of substrate translocation into lysosomes within CMA is sensitive to AD.

Since this process is sensitive to AD, it is interesting to explore the molecular mechanism of sensitivity. The mechanism is described as below and illustrated by Figure 6.

As AD progresses, the accumulation of abnormal protein increases. At this point, CMA is activated, and chaperone proteins bind to substrates, directing them towards lysosome [33, 34]. After the substrate-chaperone complex binds to LAMP2A, the chaperone complex unfolds the substrate, and the decreased expression of HSP90 accelerates substrate unfolding, thereby expediting CMA-mediated degradation. LAMP2A then forms a polymeric complex on the lysosomal membrane. GFAP regulates the stability of the complex through GTP-dependent means, and non-phosphorylated GFAP binds to LAMP2A polymeric complexes to provide stability [14, 16]. When substrate enters lysosome, EF1α dissociates from phosphorylated GFAP in the presence of GTP. This process induces conformational changes in phosphorylated GFAP, thereby attracting unphosphorylated GFAP from the LAMP2A complex and restoring LAMP2A. EEF1A1 encodes the core subunit of elongation factor 1α (EF1α). HSP90AB1 is a member of the HSP90 chaperone protein family, stabilizing proteins in the correct folded structure, but also participating in protein translocation and degradation [30]. Agaraberes et al. [35] identified HSP90 as a companion/co-companion complex member of CMA. In cellular and mouse models, the inhibition of HSP90 promotes the clearance of abnormal proteins [31]. HSP90 is believed to have the potential to unfold substrates that are folded within the complex on the lysosomal membrane [32]. Therefore, inhibiting the folding activity of HSP90 facilitates the transport of unfolded proteins and makes substrate proteins more accessible to lysosome [32]. Figure 6 illustrates this process.

The above synergistic mechanism collectively expedites the transportation of substrate into lysosome, consequently enhancing the efficiency of CMA.

However, lysosomal function has been proven to be impaired in AD. Microtubule-associated protein tau (MAPT), which encodes tau protein, damages lysosomal function through various pathways, leading to lysosomal enlargement, dysfunction, and rupture. Additionally, the regulation of genes such as TMEM106B can directly impact brain lysosomal function. Thus, even if the process of substrate entry into lysosomes is facilitated, if the lysosomes are incapable of degradation, the entire process of CMA is still inhibited. This article is primarily limited to identifying the sensitivity of the substrate entry process into lysosomes to AD, and a comprehensive assessment is required to determine whether the entire CMA process is stimulated or suppressed.

Shortcomings in this research:

1. After conducting computational experiments, this study lacks biological experiments to support its theories. The absence of experimental validation may lead to discrepancies in the results. To address this issue, efforts were made in data preprocessing to ensure the authenticity and reliability of the computational outcomes. The initial dataset GSE15222 had already undergone noise reduction operations to maintain consistency in gene expression across different samples. In this study, noise was further reduced in GSE15222 through z-score normalization, eliminating the impact of experimental errors. Additionally, the average values of different probes for the same gene were taken to minimize noise. Finally, by integrating literature analysis, theory and computational results were combined to arrive at the analysis conclusions.
2. This paper did not opt for protein expression data for experimental validation. Undoubtedly, protein data is more precise and could result in more accurate findings. However, due to the challenges in obtaining protein expression data and the current incapability of the team to experimentally acquire such data, gene expression data was chosen for the study. Furthermore, the advantage of AI calculations lies in their ability to analyze large volumes of data, making gene expression data beneficial in its own right.
3. During the experimental process, this study did not consider all genes involved in the CMA process, but focused only on those genes that facilitate substrate entry into lysosomes (because these genes were found to be extremely sensitive to AD in the calculation results). Therefore, the entire CMA process may be influenced by other factors, such as tau pathology leading to impaired lysosomal function, ultimately potentially inhibiting CMA.

## CONCLUSION

AD is a brain disorder that gets worse over time. It’s characterized by changes in the brain that lead to deposits of abnormal proteins. The aggregation of abnormal proteins leads to proteotoxicity and neuronal dysfunction. CMA is a lysosomal pathway of proteolysis that is responsible for the degradation of cytosolic proteins, and it contributes to cellular quality control through the removal of damaged or malfunctioning proteins.

The network responsible for substrate translocation into lysosomes during CMA includes key proteins such as GFAP, LAMP2A, HSP90AB1, and EEF1A1. These proteins, encoded by their respective genes, play a crucial role in facilitating the entry of substrates into the lysosome for degradation. As AD progresses, the increased accumulation of substrates triggers the activation of this specific phase of CMA, directing substrates towards lysosomal degradation through a sequence of steps. The initial phase involves the preparation for substrate delivery. HSP90AB1 acts to unfold the substrate, making it primed for delivery to the LAMP2A complex. Although HSP90AB1 functions as an inhibitory protein by binding to the substrate and potentially hindering the unfolding process, its downregulation is advantageous for facilitating substrate unfolding. This initial action thus enhances substrate readiness for entry into the LAMP2A complex. The subsequent phase encompasses the actual delivery process. Here, LAMP2A and GFAP collaborate to form a translocation complex that associates with the substrate, enabling its transport to the lysosome. The final step marks the completion of delivery. EEF1A1’s encoded protein separates GFAP from the complex, thereby resetting LAMP2A to its original state, ready for the next cycle of substrate degradation. This organized progression underscores the intricate and coordinated mechanism of substrate translocation into lysosomes, crucial for combating the proteotoxicity associated with AD progression.

It has been noted that the process of substrate translocation into lysosomes during CMA exhibits increased sensitivity to the progression of AD. Initially, HSP90AB1’s downregulation aids in substrate unfolding, thereby hastening its delivery to the lysosome. This reduction in HSP90AB1 levels indicates an excess accumulation of substrate proteotoxicity, which in turn elevates the risk of AD in patients. This relationship is highlighted in Figure 5B, where lower HSP90AB1 expression correlates with more severe dementia. During the delivery phase of this CMA process, GFAP and LAMP2A synergize to form a complex that facilitates substrate transport. A pronounced upregulation of both proteins suggests enhanced delivery efficiency, spurred by the buildup of proteotoxicity. This observation is reflected in Figure 5A, 5D, demonstrating that higher expressions of GFAP and LAMP2A are associated with increased dementia severity. At the culmination of the CMA process, EEF1A1 disengages the complex, returning CMA to its baseline state in preparation for subsequent delivery cycles. Elevated EEF1A1 expression implies that the rapid dissociation of this complex, driven by proteotoxic accumulation, boosts the overall efficiency of CMA. This dynamic is captured in Figure 5C, where higher levels of EEF1A1 are linked to greater dementia. Overall, the CMA process operates cohesively, with the proteins involved demonstrating enhanced synergy throughout the substrate delivery phase. This augmented cooperation is evidenced in Figure 4, which shows that the correlation among CMA proteins strengthens in line with AD progression. Here, the degree of protein synergy within the CMA network serves as a measure of its collective functional efficacy.

The process of substrate translocation into lysosomes within CMA exhibits not just a sensitivity to AD progression but also a preferential response to the disease, as discussed in “Drill out the set *S*_4_ that consists of the genes causing molecular network sensitive to AD” section.

The cooperative interaction among the proteins involved in this specific phase of CMA acts as a significant biomarker for AD, as demonstrated in Figure 7. For instance, when proteins such as GFAP and LAMP2A are simultaneously upregulated beyond certain thresholds, there exists a near-certainty of AD risk for the patient (Figure 7A). The complex formed by GFAP and LAMP2A plays a critical role in the degradation of substrates within lysosomes. Elevated expressions of these proteins indicate an excess accumulation of abnormal proteins awaiting degradation, leading to a heightened risk of AD. It’s important to underscore that while individual proteins offer limited biomarker utility, the collective functionality of all proteins involved in this CMA process provides a robust biomarker, reflecting the integrated nature of their action.

Furthermore, the insights presented stem from an extensive analysis of over 10,000 genes and 363 patients, utilizing two distinct AI algorithms. One algorithm was dedicated to pinpointing genes with heightened sensitivity to AD, while the other focused on identifying the molecular networks particularly responsive to the disease. Through this dual approach, the critical role of the substrate translocation process during CMA in relation to AD was elucidated.

## MATERIALS AND METHODS

### Data source and organization

#### Original data

The gene expression datasets used in this paper were downloaded from the Gene Expression Omnibus (GEO) database (https://www.ncbi.nlm.nih.gov/geo/browse/).

**Figure 8:**
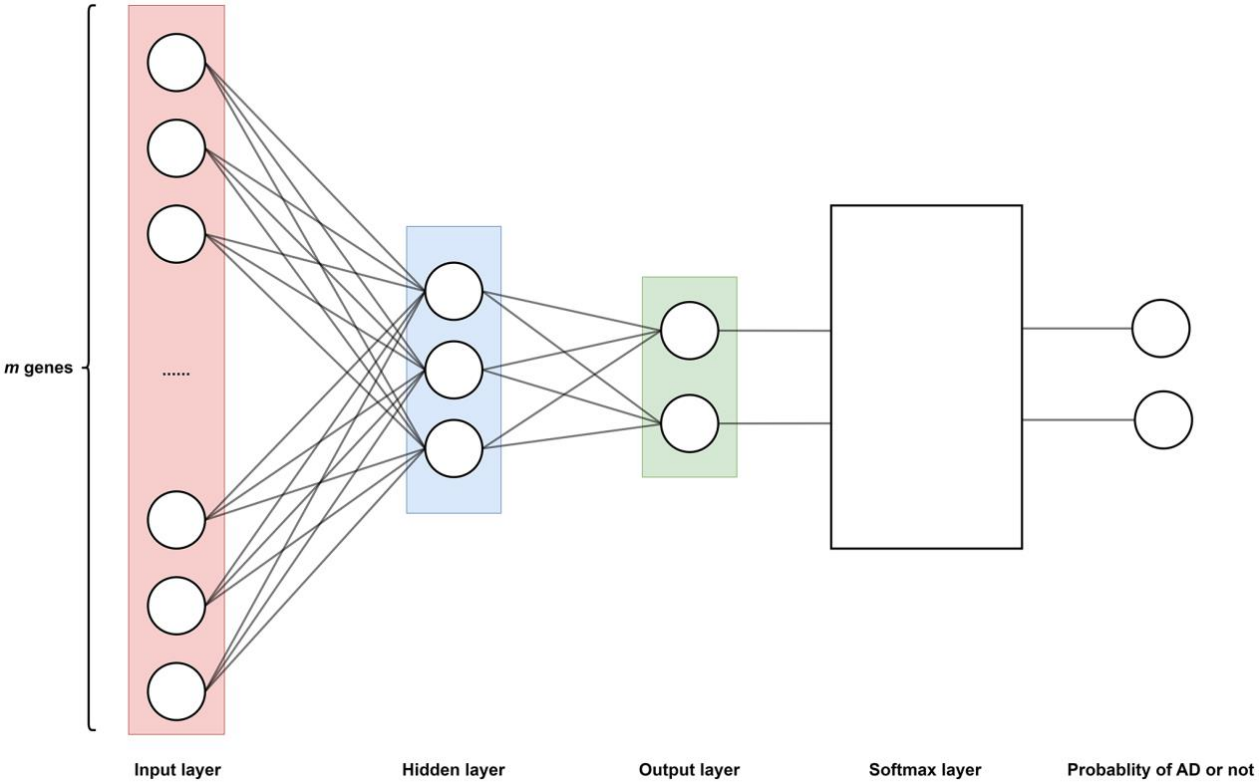
**The neural network model determines the function *f* as shown in Equation 3, which is divided into an input layer (*m* neurons, *m* = 16782), a hidden layer (3 neurons), and an output layer (2 neurons).** Where each neuron corresponds to a gene expression in a certain sample, thus a total of *m* genes corresponds to *m* neurons. Sigmoid function as an activation function in hidden layers. A Softmax layer is added to the output layer to transform the output of output layer to probability. Therefore, the output of function *f* represents the probability of having AD or not.

To train the AI model of this paper (Figure 8), the gene expression profile GSE15222 is selected. GSE15222 is based on the GPL2700 platform. GSE15222 consists of 363 samples, in which 187 control patients and 176 AD patients are included. In GSE15222, 16782 genes are included. For every patient, the expression levels of 16782 genes are sampled, where the data of different probes are combined into one item using their average if the probes correspond to a same gene. So, the total original data is 363 × 16782.

To explore the relationship between gene expression level and clinical indicator (Figure 5), GSE1297 is used in this paper, which consists of 9 controls and 22 AD subjects. The 31 subjects include data of clinical indicator [36], so the clinical analysis benefits from these data. It should be noted that, in GSE5281 and GSE1297, there are also instances of multiple probes corresponding to a single gene. Therefore, the mean value of the probes is used as the expression of the gene for these two datasets as well.

To explore the biomarker of AD (Figure 7), GSE5281 is used, in which 87 AD samples and 74 normal samples are included.

#### The normalization of original data

For every patient (or a sample, or a subject), 16782 genes are sampled, then 16782 data are obtained. And these data form a sequence. Let ZScore normalization algorithm act on the sequence. Then the normalized sequence is the output.

#### The organization of the normalized data

After data are normalized, all data are organized as the following matrix.


$$X=\left(\begin{array}{ccc} x_{11} & \cdots & x_{1n}\\ \vdots & \ddots & \vdots \\ x_{m1} & \cdots & x_{mn} \end{array}\right)$$

In the above matrix, “*m*” represents the number of genes, and “*n*” represents the number of samples, including both patients and controls. “*x*_ij_” represents the expression level of the *i* − *th* gene expression which is sampled from the *j* − *th* patient.

In this paper, *n* = 363 *m* = 16782. That is, all of the original data are sampled from 363 patients and 16782 genes are tested.

Let ${\vec{s}}_{j}$ denote the *j* − *th* column vector. That is,


$${\vec{s}}_{j}\;=\left(x_{1j},\;x_{2j},\;\;\ldots \;,\;x_{ij},\;\;\ldots \;,\;x_{mj}\right)$$

The column vector ${\vec{s}}_{j}$ is a data sequence, in which all data are sampled from the *j* − *th* patient and *m* genes are sampled totally.

Then all of the gene data can be represented as following format also.


$$X=\left({\vec{s}}_{1}\;\;\ldots \;\;{\vec{s}}_{j}\;\;\ldots \;\;{\vec{s}}_{n}\right)\;\;\;\text{(Eq}\text{. 1)}$$

In Eq. 1, all data are organized by samples (patients), every patient corresponds to a column vector.

Let ${\vec{g}}_{i}$ denote the *i* − *th* line vector. That is,


$${\vec{g}}_{j}\;=\left(x_{i1},\;x_{j2},\;\;\ldots \;,\;x_{ij},\;\;\ldots \;,\;x_{in}\right)$$

The line vector ${\vec{g}}_{i}$ is a data sequence, in which all data corresponds to the *i* − *th* gene, and they are sampled from different patients.

Then all of the gene data can be represented as following format too.


$$X=\left(\begin{array}{c} {\vec{g}}_{1}\\ \vdots \\ {\vec{g}}_{i}\\ \vdots \\ {\vec{g}}_{m} \end{array}\right)\;\;\;\text{(Eq}\text{. 2)}$$

In Eq. 2, all data are organized by genes, every gene corresponds to a line vector.

### The method to identify the genes sensitive to AD

The aim of this section is to design a machine learning method to identify the genes sensitive to AD.

To reach the aim, the relationship between genes and AD is trained out as a mathematics function *y* = *f*(*x*), where *x* denotes the expression level of a gene and *y* denotes the probability of a patient having the risk of AD.

Then, derivative *f*′(*x*) measures the degree of sensitivity to AD. If value *f*′(*x*) is big, the little change of input data *x* will lead to a significant change of output data *y*.

Because many genes are related to AD, the function *y* = *f*(*x*) is updated as multivariate function *y* = *f*(*x*_1_, *x*_2_, …, *x_m_*), where (*x*_1_, *x*_2_, …, *x_m_*) represents the expression level of *m* genes respectively.

Then, the derivative *f*′(*x*) is updated as partial derivatives $\frac{\partial f}{\partial x_{1}},\;\frac{\partial f}{\partial x_{2}},\;\;\ldots \;\;,\frac{\partial f}{\partial x_{m}}.$ Every partial derivative measures the sensitivity degree of every gene to AD. For example, if the absolute value $\left|\frac{\partial f}{\partial x_{1}}\right|$ is bigger than $\left|\frac{\partial f}{\partial x_{2}}\right|,$ the first gene is more sensitive than the second gene.

The following steps are to train out the multivariate function *y* = *f*(*x*_1_, *x*_2_,…, *x*_m_), where the function *f* is realized by a neural network.

Step 1. Build and train a neural network (Figure 8).

Input data:* n* = 363 samples (or patients). For every patient, *m* = 16782 genes are sampled and generate the expression level *x*_1_, *x*_2_, …, *x*_m_ respectively. All these data are from database GSE15222.

Training neural network: The model of the neural network is illustrated as Figure 8. This model comprises distinct layers: input, hidden, and output.

The input layer holds *m* neurons, and corresponds to *m* input data *x*_1_, *x*_2_, …, *x*_*m*_, which is the expression level of *m* genes respectively. Data *x*_1_, *x*_2_, …, *x*_*m*_ are sampled from a same patient. Totally, *n* patients and *m* genes are used for training.

The hidden layer comprises three neurons. Every neuron is activated by a sigmoid function.

The output layer consists of two neurons. The output data of this layer traverses through the Softmax layer, where the Softmax layer yields the probability of patients having a risk of AD.

In sum, the model of neural network is the realization of multivariate function *y* = *f*(*x*_1_, *x*_2_, …, *x*_*m*_). And the function *f* is realized by the hidden layer, and the probability of AD risk yields by the Softmax layer.

Output of neural network: The probability of AD risk is the output. That is, for the input data sampled from a patient, his probability of AD risk will be calculated by the neural network.

In sum, the neural network is the realization of function *y* = *f*(*x*_1_, *x*_2_, …, *x*_*m*_). After training, the function is represented by the neural network. The training process and results of the neural network are detailed in Supplementary Materials.

Step 2. Calculate the partial derivatives of all genes.

Input data: For a given patient, such as the *j* − *th* patient, the expression levels of *m* genes are sampled, these data form a vector ${\vec{s}}_{j}=f(x_{1j},\;x_{2j},\;\ldots ,\;x_{ij},\;\ldots ,\;x_{mj}),$ where *x*_*ij*_ denotes the expression level of *i* − *th* gene and ${\vec{s}}_{j}$ represents the data of all genes sampled from the *j* − *th* patient. Vector ${\vec{s}}_{1},\;{\vec{s}}_{2},\;\ldots \;,\;{\vec{s}}_{n}$ form a set of input data, which is the domain of function *f*.

Calculation of the probability of AD risk: Vector ${\vec{s}}_{j}$ is input of the function *f* (i.e., the above neural network), the probability of the *j* − *th* patient having the risk of AD will be output, and labeled as *y*_*j*_. That is,


$$y_{j}=f({\vec{s}}_{j})=f\left(x_{1j},\;x_{2j},\;\ldots \;,\;x_{ij},\;\ldots \;,\;x_{mj}\right)\;\;\;\text{(Eq}\text{. 3)}$$

Output of partial derivatives: Since function *f* is known, the partial derivative $d_{ij}=\frac{\partial f}{\partial x_{ij}}$ can be calculated, where *d_ij_* denotes the value of partial derivative at the data *x*_ij_. That is, for the *i* − *th* gene, *d_ij_* denotes the value of partial derivative at the data sampled from the *j* − *th* patient. Then the following matrix is output.


$$D=\left(\begin{array}{ccc} d_{11} & \cdots & d_{1n}\\ \vdots & \ddots & \vdots \\ d_{m1} & \cdots & d_{mn} \end{array}\right)=\left(\begin{array}{ccc} \frac{\partial f}{\partial x_{11}} & \ldots & \frac{\partial f}{\partial x_{1n}}\\ \vdots & \ddots & \vdots \\ \frac{\partial f}{\partial x_{m1}} & \ldots & \frac{\partial f}{\partial x_{mn}} \end{array}\right)\;\;\;\text{(Eq}\text{. 4)}$$

Every line of the matrix corresponds to a gene, and every data in the line represents the value of partial derivative obtained from different patients.

Every column of the matrix corresponds to a patient, and every data in the column represents the value of partial derivative of different gene.

Step 3. Calculate the average of the absolute value of partial derivatives for every gene.


$${\bar{d}}_{i}=\frac{1}{n}\sum_{j=1}^{n}\left|d_{ij}\right|\;\;\;\text{(Eq}\text{.}4^{\prime }\text{)}$$

Where, for the *i* − *th* gene, ${\bar{d}}_{i}$ denotes the average of the absolute value of partial derivatives, and *i* = 1, …, *m*, *j* = 1, …, *n*. That is, from *n* patients, the average value ${\bar{d}}_{i}$ is calculated.

Step 4. Sort all value ${\bar{d}}_{i}$ by descending order (*i* = 1, …, *m*).

The value ${\bar{d}}_{i}$ measures the degree of sensitivity to AD holding view of statistics. If the *i* − *th* gene holds big ${\bar{d}}_{i}$, a little change of its expression level will lead to big change of the probability of AD risk. The bigger the value ${\bar{d}}_{i}$, the more sensitive the *i* − *th* gene. That is, the *i* − *th* gene is sensitive to AD if it holds big ${\bar{d}}_{i}$.

The detailed introduction of the above method is listed at Supplementary Materials, and its calculation flow is shown in Algorithm 4-1 (Table 4).

**Table 4:** The calculation of derivative.

Algorithm 4-1 The calculation of derivative
** Inputs:** The expression of all genes in each sample
** Outputs:** A gene sequence sorted based on gene sensitivity to AD
** Steps:**
** Step 1.** Building neural network and train out function *f*.
Mathematics model: Eq. 3
Training data: 80% of all samples.
Test data: 20% of all samples.
Optimization function: stochastic gradient descent (SGD).
Iteration number: 100.
Learning rate: 0.0001.
Validation method: ten-fold cross-validation.
** Step 2.** Calculate the partial derivatives of all genes.
The model of calculation is shown at Eq. 4, and detail is listed at Supplementary Materials.
** Step 3.** Calculate the average of partial derivative of every gene
The calculation formula is listed at Eq. 4′.
** Step 4.** Sort all genes by their average of partial derivative.

### The method to identify the genes sensitive to AD through molecular network

Molecular networks perform their specific biological functions. For example, CMA performs the function of transporting substrates to lysosomes for degradation. If the development of AD stimulates the activity of CMA, then for a patient, the probability of having AD can be reflected through CMA. From a mathematical perspective, the relationship between CMA and AD can be described by a function ‘*f*’, such that *y* = *f*(*CMAgenes*). *CMAgenes* represents the expression levels of all genes within the CMA network, and ‘*y*’ represents the probability of the patient having an AD risk caused by CMA network. If the removal of a specific gene from CMA leads to a significant change of probability, it can be inferred that this gene is sensitive to AD and has significant contribution to CMA network. That is, the gene causes CMA sensitive to AD.

Guiding by the above idea, the following method is proposed to identify genes causing molecular networks to AD.

Step 1. Build neural networks and train out mathematics functions between the molecular network and the probability of AD risk.

For example, CMA network consists of the genes GFAP, LAMP2A, EEF1A1, and HSP90AB1, the following function can be trained using the method of Figure 8.


$$y=f_{1}(x_{1},x_{2},x_{3},x_{4})$$

Where *x*_1_, *x*_2_, *x*_3_, and *x*_4_ represent the expressions of GFAP, LAMP2A, EEF1A1, and HSP90AB1, respectively, and ‘*y*’ represents the AD risk probability.

The domain of function *f*_1_ is the gene expression levels of four genes of CMA. So, the function reflects the relationship between CMA and AD.

Step 2. For a given gene, measure its contribution to molecular network.

For example, if GFAP is excluded from CMA, another function *w* = *f*_2_(*x*_2_, *x*_3_, *x*_4_) will be trained out.

Let Δ = *y* – *w*. Then, the difference Δ measures the contribution of GFAP to network CMA. The bigger the difference, the more significant the contribution.

In fact, GFAP also participates in other molecular networks and plays different roles, and leads to other values similar to Δ. Calculate the average value of these data, and denoted by $\bar{\text{Δ}}.$ Then, the bigger $\bar{\text{Δ}},$ the more significant the contribution caused by GFAP. The bigger $\bar{\text{Δ}},$ the more important the role of GFAP within a network.

Similarly, for any gene, its contribution can be estimated.

Step 3. Shapley’s method is used to estimate the contribution of a gene to molecular network.

To calculate the average $\bar{\text{Δ}}$ of any gene, Shapley’s method is proposed in this paper.

The theory of Shapley’s method: Shapley’s method comes from game theory, and Shapley value serves as a metric for fairly distributing rewards among a set of participants who contribute to an outcome [37]. Shapley’s method outputs Shapley value, its computation method is presented by Lundberg and Lee [24], and the detail of computation is listed in the Supplementary Materials. In the Supplementary Materials, the Shapley value is labeled as *φ*. In game theory, the bigger the value *φ* held by a participant, the more significant the effect on game caused by the participant.

The application of Shapley’s method in this paper: In this paper, a molecular network corresponds to a game of Shapley’s method, every gene corresponds to a participant of game. And value Δ corresponds to a participant’s contribution to the game, which is measured by Shapley value *φ*. Thus, the Shapley value *φ* counts how much a gene influences AD through all possible networks. A larger *φ* indicates that the gene can have a greater impact on AD across different gene networks.

Using Shapley’s method, the average of Shapley value *φ* can be estimated. Therefore, the average $\bar{\text{Δ}}$ can be estimated by the average of *φ*. That is, the contribution of a gene to molecular network can be estimated by the average of *φ*.

The Shapley’s calculation method is shown in Algorithm 4-2 (Table 5).

**Table 5:** Shapley calculation method.

Algorithm 4-2 Shapley calculation method
** Inputs:** The expression of all genes in each sample
** Outputs:** A gene sequence sorted based on gene contribution (Shapley’s value) to AD
** Steps:**
** Step 1.** For the *i* − *th* gene, calculate Shapley value at *j* − *th* sample.
Where, the value is denoted by *φ_ij_*, *i* = 1, …, *m*, *j* = 1, …, *n*.
The calculation procedure is described in Supplementary Materials.
** Step 2.** Calculate the average of Shapley’s value.
Let $shapley_{i}=\frac{1}{n}\;\sum_{j=1}^{n}\left|\varphi_{ij}\right|,\;i=1,\;\ldots \;,\;m.$
The *shapley*_i_ is the average Shapley value, it represents the contribution of the *i* − *th* gene to molecular network. The bigger the value, the more significant the contribution.
** Step 3.** Sort genes in descending order by their Shapley values.

### Enrichment analysis

The sensitivity of each individual’s genes to AD and the sensitivity of given genes to AD through the network are calculated in the above two sections. The intersection of the results from both calculations *S*_5_ (1575 genes) simultaneously possesses these two characteristics. Thus, networks sensitive to the progression of AD are hidden within the set *S*_5_. These sensitive networks cannot be visually recognized because 1575 is too vast an amount of information for human perception. Therefore, functional enrichment analysis aids in identifying molecular networks highly correlated with AD. The intersection set of two significance rankings *S*_5_ was analyzed for GO and KEGG pathways by the ‘clusterProfiler’ package enrichment function in the R software as a way to screen for the most significant networks for AD *p* < 0.05, was considered as the cut-off criterion. For the results of the GO analysis, the functional network was screened with the optimal genes ranked by the above algorithms.

### Protein-protein interaction network analysis

The PPI network was employed to further identify the core genes in the functional network. Gene interactions with known or predicted direct (physical) and indirect (functional) PPIs in *S*_6_ were retrieved using the search tool (STRING version 11.5). The significant nodes were identified using the betweenness centrality algorithm of the CytoNCA [38] plug-in, as shown in Equation 5.


$$g(\upsilon )=\sum_{s\neq \upsilon ,s\neq t}\frac{\sigma_{st}(\upsilon )}{\sigma_{st}}\;\;\;\text{(Eq}\text{. 5)}$$

In the context of considering each gene in the network as a node, the notation *σ_st_*(*υ*) represents the number of shortest paths from a specific node *s* (a particular gene) to another node *t* (another gene) passing through node *υ* (yet another gene). On the other hand, *σ_st_* represents the number of shortest paths from node *s* to node *t*. Consequently, *g*(*υ*) represents the node (gene) with the highest number of connections in the network, commonly referred to as the hub node. Betweenness centrality plays a crucial role in the analysis of biological networks, and betweenness centrality, in particular, is frequently applied to mammalian transcriptional regulatory networks to reveal potential biological features [39]. The *S*_6_ set is brought into this algorithm to analyze the importance of CMA related genes, which is calculated and analyzed to get about the CMA network *S*_7_.

### Statistical analysis

We used boxplots to count the expression of *S*_7_ in each of the three datasets. GSE15222 and GSE5281 show the expression of the *S*_7_ gene in the control group versus the AD group, respectively. GSE1297 shows the expression of genes according to the degree of dementia. In the correlation analysis, cosine similarity was used to describe the expression trends of the four genes because the GSE15222 data were normalized beforehand. The results of the correlation analysis are shown in the form of heatmaps. Finally, in the analysis with clinical indicators, the relationship between clinical and gene expression was calculated using the dataset GSE1297, and the trend of gene variation was verified. The specific procedure used univariate linear regression to calculate the relationship between MMSE, NFT and expression values.

### CMA validation model

A diagnostic model was constructed by applying a support vector machine (SVM) in Python (version 3.8) using the “sklearn” package. The model is able to distinguish between AD and normal samples by different combinations of important genes. The samples of GES5281 dataset were randomly assigned to the training set (80%) and the test set (20%). The model was used for validation of the screened genes and further exploration of Alzheimer’s disease.

### Data availability statement

The expression data GSE1297 (https://www.ncbi.nlm.nih.gov/geo/query/acc.cgi?acc=GSE1297), GSE15222 (https://www.ncbi.nlm.nih.gov/geo/query/acc.cgi?acc=GSE15222) and GSE5281 (https://www.ncbi.nlm.nih.gov/geo/query/acc.cgi?acc=GSE5281) used in this study are available in the GEO database. All data obtained for this study are included in the article and further inquiries can be made by contacting the corresponding author. The code used in this study is available from the corresponding author upon reasonable request.

## Supplementary Materials

Supplementary Methods

Supplementary Figures

Supplementary File 1

Supplementary File 2

Supplementary File 3

Supplementary File 4

Supplementary File 5

Supplementary File 6
