# Supplementary material for: Sensitivity of substrate translocation in chaperone-mediated autophagy to Alzheimer’s disease progression
Source: Aging (Albany NY). 2024 May 23;16(10):9072–105. doi: 10.18632/aging.205856 (PMC11164475; doi:10.18632/aging.205856)
Supplement: Supplementary Methods [file aging-16-205856-s001.pdf]

SUPPLEMENTARY METHODS

Data source and organization

Original data

The gene expression datasets used in this paper were downloaded from the Gene Expression Omnibus (GEO) database (https://www.ncbi.nlm.nih.gov/geo/browse/).

To train the AI model of this paper (Figure 8), the gene expression profile GSE15222 is selected. GSE15222 is based on the GPL2700 platform. For every patient, the expression levels of 16782 genes are sampled. So, the total original data is 363 × 16782.

The normalization of original data

For every patient (or a sample, or a subject), 16782 genes are sampled, then 16782 data are obtained. And these data form a sequence. Let ZScore normalization algorithm act on the sequence. Then the normalized sequence is the output.

The organization of the normalized data

After data are normalized, all data are organized as the following matrix.

$$X = \begin{pmatrix} x_{11} & \cdots & x_{1n} \\ \vdots & \ddots & \vdots \\ x_{m1} & \cdots & x_{mm} \end{pmatrix}$$

In the above matrix, "m" represents the number of genes, and "n" represents the number of samples, including both patients and controls. "x_{ij}" represents the expression level of the $i-th$ gene expression which is sampled from the $j-th$ patient.

In this paper, n = 363 m = 16782. That is, all of the original data are samples from 363 patients and 16782 genes are tested.

Let $\vec{s}_j$ denotes the $j-th$ column vector. That is,

$$\vec{s}_j = (x_{1j}, x_{2j}, \ldots, x_{ij}, \ldots, x_{mj})$$

The column vector $\vec{s}_j$ is a data sequence, in which all data are sampled from the $j-th$ patient and total m genes are sampled.

Then all of the gene data can be represented as following format also.

$$X = (\vec{s}_1 \quad \ldots \quad \vec{s}_j \quad \ldots \quad \vec{s}_n) \qquad \text{(Eq. 1)}$$

In Eq. 1, all data are organized by samples (patients), every patient corresponds to a column vector.

Let $\vec{g}_i$ denote the $i-th$ line vector. That is,

$$\vec{g}_i = (x_{i1}, x_{i2}, \ldots, x_{ij}, \ldots, x_{in})$$

The line vector $\vec{g}_i$ is a data sequence, in which all data corresponds to the $i-th$ gene, and they are sampled from different patients.

Then all of the gene data can be represented as following format too.

$$X = \begin{pmatrix} \vec{g}_1 \\ \vdots \\ \vec{g}_i \\ \vdots \\ \vec{g}_m \end{pmatrix} \qquad \text{(Eq. 2)}$$

Training of neural network models

Input data

n = 363 samples (or patients). For every patient, m = 16782 genes are sampled and generate the expression level $x_1, x_2, \ldots, x_m$ respectively. All these data are from database GSE15222.

Training neural network

The model of the neural network is illustrated as Figure 1. This model comprises distinct layers: input, hidden, and output.

The input layer holds m neurons, and corresponds to m input data $x_1, x_2, \ldots, x_m$, which is the expression level of m genes respectively. Data $x_1, x_2, \ldots, x_m$ are sampled from a same patient. Totally, n patients and m genes are used for training.

The hidden layer comprises three neurons. Every neuron is activated by a sigmoid function.

The output layer consists of two neurons. The output data of this layer traverses through the Softmax layer, where the Softmax layer yields the probability of patients having a risk of AD [1].

In sum, the model of neural network is the realization of multivariate function $f(x_1, x_2, \ldots, x_m)$. And the function f is realized by the hidden layer, and the probability of AD risk yields by the Softmax layer.

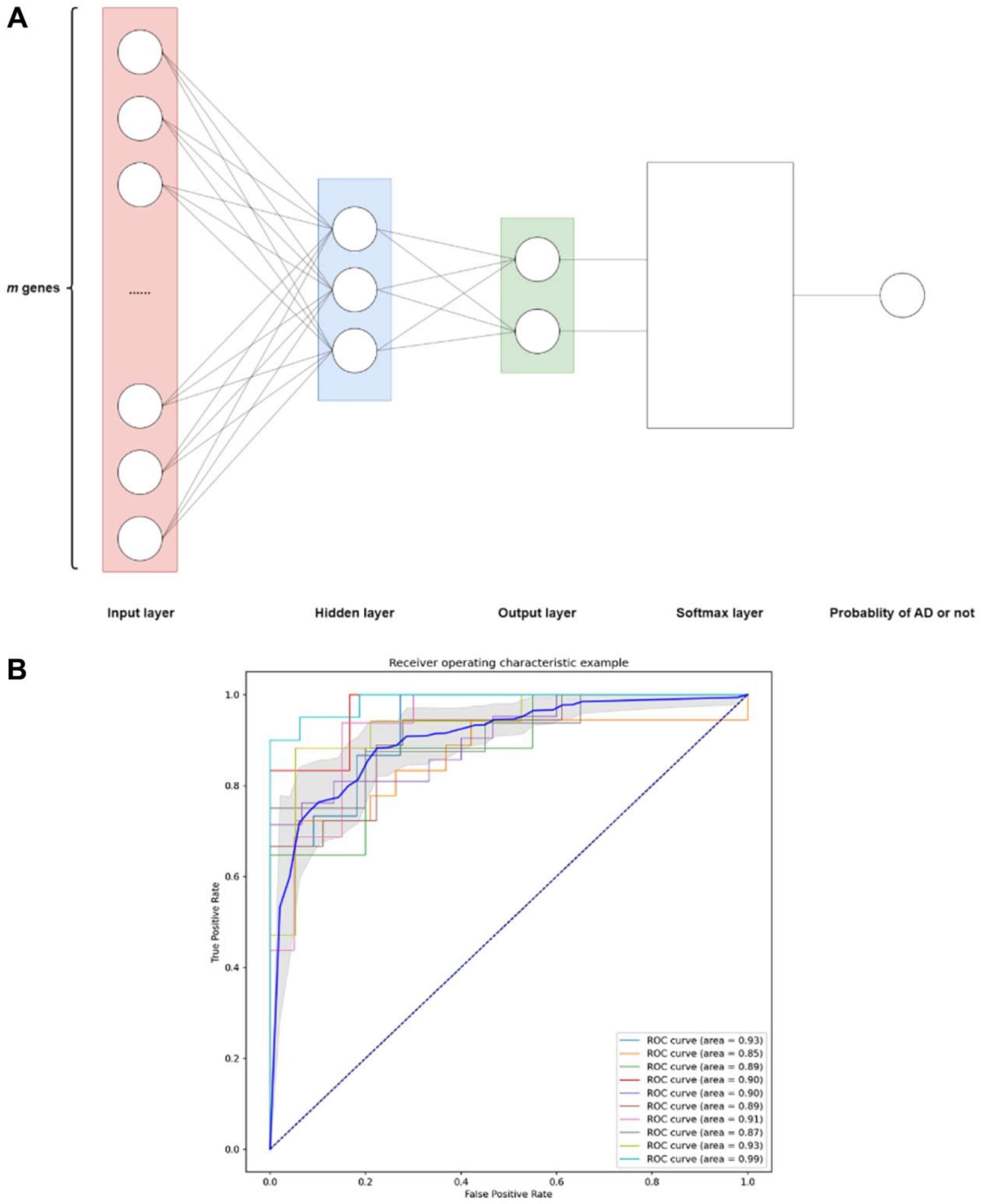

Figure 1. The schematic diagram of the computational model. (**A**) The neural network model determines the function *f* as shown in Equation 3, which is divided into an input layer (***m*** neurons, ***m* = 16782**), a hidden layer (3 neurons), and an output layer (2 neurons). Where each neuron corresponds to a gene expression in a certain sample, thus a total of *m* genes corresponds to *m* neurons. Sigmoid function as an activation function in hidden layers. A Softmax layer is added to the output layer to transform the output of output layer to probability. Therefore, the output of function *f* represents the probability of having AD or not. (**B**) ROC curve image obtained by 10-fold cross-validation. The relationship between sensitivity and specificity of the model is reflected by the curve image. The horizontal axis is the false positive rate (false alarm rate), the closer to zero the higher the accuracy rate; The vertical axis is called the true positive rate (sensitivity), and the larger it is the higher the accuracy rate. The area under the curve is called the AUC (Area Under Curve), which indicates the prediction accuracy. The higher the AUC value, that is, the larger the area under the curve, the higher the prediction accuracy.

Output of neural network

The probability of AD risk is the output. That is, for the input data sampled from a patient, his probability of AD risk will be calculated by the neural network.

In sum, the neural network is the realization of function $y = f(x_1, x_2, \ldots, x_m)$. After training, the function is represented by the neural network.

For this study, 80% (290 samples) was allocated as training data and 20% (73 samples) was allocated as testing data. The optimization process involves employing the stochastic gradient descent (SGD) algorithm with a learning rate of 0.0001. Upon 100 iterations, the function successfully converges.

In addition, ten-fold cross-validation is used to assess the performance and generalization ability of machine learning models by segmenting the dataset, training and validating it multiple times to derive reliable performance metrics. It helps to prevent overfitting, as well as to evaluate model performance under uneven data distributions, and is ultimately used to select the most suitable model for the task. The results of the ten-fold cross-validation method are shown in Figure 1B. The images show an area under the curve greater than 50%, indicating high prediction accuracy. And the model performs stably on each fold without significant performance differences, indicating that the model generalizes well and is not overfitted.

Derivative calculation method in this paper

The principle of method

When the independent variable of a function varies at a particular point, the derivative at that point is defined as the ratio of the change in the output value to the change in the independent variable, as the change in the independent variable approaches zero. Thus, the derivative of a function at a point describes the rate of change of that function near that point.

The genes related to AD hold the feature in general that its expression level will change with AD progression, and this type of gene is considered in this paper. If a gene holds the above feature, its expression level x is associated with the probability y, where y is the probability that the patient has the risk of AD. In other words, there is a function f such as $y = f(x)$. And the derivative $f'(x)$ represents the degree of sensitivity to AD progression, the bigger $f'(x)$, the more sensitive the gene. That is, a slight change in the expression level x leads to a significant change in the probability of AD risk.

Method

Let's contemplate a ternary function, denoted as f. This function takes three inputs: x, y, and z, yielding an output u. This relationship is represented by Equation 3.

$$u = f_{example}(x, y, z) \qquad \text{(Eq. 3)}$$

Consequently, the partial derivative of y at the specific point (x_0, y_0, z_0) can be articulated as follows:

$$\frac{\partial u}{\partial y}\Big|_{x=x_0, y=y_0, z=z_0} = \lim_{\Delta y \to 0} \frac{\Delta u}{\Delta y} = \lim_{\Delta y \to 0} \frac{f_{example}(x_0, y_0+\Delta y, z_0) - f_{example}(x_0, y_0, z_0)}{\Delta y} \qquad \text{(Eq. 4)}$$

Equation 4 can be understood by holding the values of x and z constant at x_0 and z_0 while allowing y to undergo a slight increment Δy around y_0. Consequently, the function $u = f_{\text{example}}(x,y,z)$ yields an increment $\Delta u = f_{\text{example}}(x_0, y_0, + \Delta y, z_0) - f_{\text{example}}(x_0, y_0, z_0)$. As Δy approaches infinitesimally small values, the ratio $\frac{\Delta u}{\Delta y}$ is referred to as the partial derivative of function f_{example} concerning variable y at the specific points x_0, y_0 and z_0.

Hence, the partial derivative of function f_{example} with respect to y signifies the rate of transformation of the function concerning the variable y at the specific coordinates (x_0, y_0, z_0). This rate of alteration indicates the extent to which y influences the outcome of the function u. A higher derivative implies that even a minor alteration in y leads to a substantial shift in the function's output, u. Conversely, the opposite holds true as well.

The neural network function is shown in Equation 5. If we substitute the function f_{example} with f, the independent variables x, y, and z will be substituted with gene expressions $x_1, x_2, \ldots, x_m$. The dependent variable becomes the estimated probability of Alzheimer's disease, denoted as y. Consequently, for a specific gene i, the partial derivative at a particular point indicates the extent to which that gene influences the probability of Alzheimer's disease. This relationship is depicted in Equation 6.

$$y = f(x_1, x_2, \ldots, x_m) \qquad \text{(Eq. 5)}$$

$$\frac{\partial \hat{y}}{\partial x_i} = \lim_{\Delta x_i \to 0} \frac{\Delta \hat{y}}{\Delta x_i} = \lim_{\Delta x_i \to 0} \frac{f(x_1, \ldots, (x_i + \Delta x_i), \ldots, x_m) - f(x_1, \ldots, x_i, \ldots, x_m)}{\Delta x_i} \qquad \text{(Eq. 6)}$$

Input data

For a given patient, such as the $j-th$ patient, the expression levels of m genes are sampled, these data form a vector $\vec{s}_j = (x_{1j}, x_{2j}, \ldots, x_{ij}, \ldots, x_{mj})$, where x_{ij} denotes the expression level of $i-th$ gene and $\vec{s}_j$ represents the data of all genes sampled from the $j-th$

patient. Vector $\vec{s}_1, \vec{s}_2, \ldots, \vec{s}_n$ form a set of input data, which is the domain of function *f*.

Calculation of the probability of AD risk

Vector $\vec{s}_j$ is input the function *f*(i.e., the above neural network), the probability of the $j-th$ patient having the risk of AD will be output, and labeled as y_j. That is,

$$y_j = f(\vec{s}_j) = f(x_{1j}, x_{2j}, \ldots, x_{ij}, \ldots, x_{mj}) \quad \text{(Eq. 7)}$$

Output of partial derivatives

Since function *f* is known, so the partial derivative $d_{ij} = \frac{\partial f}{\partial x_{ij}}$ can be calculated, where d_{ij} denotes the value of partial derivative at the data x_{ij} That is, for the $i-th$ gene, d_{ij} denotes the value of partial derivative at the data sampled from the $j-th$ patient. Then the following matrix is output.

$$D = \begin{pmatrix} d_{11} & \cdots & d_{1n} \\ \vdots & \ddots & \vdots \\ d_{m1} & \cdots & d_{mn} \end{pmatrix} = \begin{pmatrix} \frac{\partial f}{\partial x_{11}} & \cdots & \frac{\partial f}{\partial x_{1n}} \\ \vdots & \ddots & \vdots \\ \frac{\partial f}{\partial x_{m1}} & \cdots & \frac{\partial f}{\partial x_{mn}} \end{pmatrix} \quad \text{(Eq. 8)}$$

Every line of the matrix corresponds to a gene, and every data in the line represents the value of partial derivative obtained from different patients.

Every column of the matrix corresponds to a patient, and every data in the column represents the value of partial derivative of different gene.

Calculate the average of partial derivative of every gene

$$\bar{d}_i = \frac{1}{n}\sum_{j=1}^{n}\left|d_{ij}\right| \quad \text{(Eq. 9)}$$

Where, for the $i-th$ gene, $\bar{d}_i$ denotes the average of the absolute value of partial derivatives, and $i = 1, \ldots, m$, $j = 1, \ldots, n$. That is, from *n* patients, the average value $\bar{d}_i$ is calculated.

$$\bar{D} = \begin{pmatrix} \bar{d}_1 \\ \vdots \\ \bar{d}_i \\ \vdots \\ \bar{d}_m \end{pmatrix} \quad \text{(Eq. 10)}$$

Sort all genes by the average of partial derivative

Sort all genes in descending order of the average of partial derivative. The output is the gene orders after sorting.

Shapley calculation method in this paper

Shapley is one of the important calculations in this paper and therefore will be described in detail. Shapley value is a mathematical concept in game theory and was introduced by Lloyd Stowell Shapley in 1951 [2].

The principle of method

A molecular network performs its corresponding biological function. For example, CMA delivers substrate to the lysosome to degrade. With the AD progression, the aggregation of abnormal proteins becomes heavier, the function of delivery is stimulated, and CMA becomes active. Then, for a given patient, his probability having AD risk is reflected by CMA. Holding a view of mathematics, there is a function *f* such that $y = f(CMAgenes)$, where *CMAgenes* represents the expression levels of all genes of CMA, and *y* is the probability that the patient has the risk of AD caused by network *CMA*. If the change of expression levels of genes in CMA leads to a significant change of probability, it can be deduced that CMA is sensitive to AD. Then, it is useful to use machine learning to train out the function *f*.

Method

Guided by the above idea, the following methods are proposed to identify genes causing molecular networks to AD.

For example, the molecular network CMA consists of gene GFAP, LAMP2A, EEF1A1 and HSP90AB1. Using machine learning, the function $y = f_1(x_1, x_2, x_3, x_4)$ will be trained out, where x_1, x_2, x_3, x_4 represents the expression level of GFAP, LAMP2A, EEF1A1 and HSP90AB1 respectively, and y represents the probability of AD risk. The domain of function f_1 is the gene expression levels of four genes of CMA. So, the function reflects the relationship between CMA and AD.

If GFAP is excluded from CMA, the other function $w = f_2(x_2, x_3, x_4)$ will be trained out. Then, the difference of probability $\Delta = y - w$ measures the effect of GFAP on AD through network CMA. And the bigger the value Δ, the more significant the effect of GFAP on AD.

In fact, GFAP also participates in other molecular networks and plays different roles, leading to other values similar to Δ. Calculate the average value of these data, and denoted by $\bar{\Delta}$. Then, the bigger $\bar{\Delta}$, the more significant the contribution caused by GFAP. The bigger $\bar{\Delta}$, the more important the role of GFAP within a network.

Similarly, for any gene, its contribution can be estimated. Shapley value is used to estimate the contribution of a gene to molecular network. To calculate the average $\overline{\Delta}$ of any gene, Shapley value is proposed in this paper.

The theory of Shapley's method

Shapley's method comes from game theory, and Shapley value serves as a metric for fairly distributing rewards among a set of participants who contribute to an outcome. Shapley's method outputs Shapley value, its computation method is shown in Equation 11, where φ_i represents the Shapley value of the *i – th* gene, which also indicates the sensitivity of the *i – th* gene to AD after passing through the molecular network. The Shapley values in this paper is approximated in this study using the Shap framework proposed by Lundberg and Lee.

$$\varphi_i = \sum_{\boldsymbol{S}\subseteq \boldsymbol{F}-\{g_i\}} \frac{|\boldsymbol{S}|!(|\boldsymbol{F}|-|\boldsymbol{S}|-1)!}{|\boldsymbol{F}|!}\left(f_{\boldsymbol{S}\cup g_i}(\boldsymbol{S}\cup g_i) - f_{\boldsymbol{S}}(\boldsymbol{S})\right) \quad \text{(Eq. 11)}$$

Input data

The expression of all genes in each sample.

Output of Shapley values

Similar to the computation of partial derivatives, using the Shap framework, the Shapley value φ_{ij} can be estimated. Where φ_{ij} denotes the Shapley value at the data x_{ij}. That is, for the *i – th* gene, φ_{ij} denotes the Shapley value at the data sampled from the *j – th* patient. Then the following matrix is the output.

$$D = \begin{pmatrix} \varphi_{11} & \cdots & \varphi_{n1} \\ \vdots & \ddots & \vdots \\ \varphi_{1m} & \cdots & \varphi_{mn} \end{pmatrix} \quad \text{(Eq. 12)}$$

Every line of the matrix corresponds to a gene, and every data in the line represents the Shapley values obtained from different patients.

Every column of the matrix corresponds to a patient, and every data in the column represents the Shapley values of different gene.

Calculate the average of Shapley values of every gene

$$\bar{\varphi}_i = \frac{1}{n}\sum_{j=1}^{n}|\varphi_{ij}| \quad \text{(Eq. 13)}$$

Where, for the *i – th* gene, $\bar{\varphi}_i$ denotes the average of the absolute value of partial derivatives, and $i = 1, \ldots, m$, $j = 1, \ldots, n$. That is, from n patients, the average value $\bar{\varphi}_i$ is calculated.

$$\overline{D} = \begin{pmatrix} \bar{\varphi}_1 \\ \vdots \\ \bar{\varphi}_i \\ \vdots \\ \bar{\varphi}_m \end{pmatrix} \quad \text{(Eq. 14)}$$

Output

Sort all genes in descending order of the average Shapley values. The output is the genes orders after sorted.

The method for estimating Shapley values using Shap

The kernel SHAP proposed by Lundberg and others combines the Local Interpretable Model-agnostic Explanations (LIME) algorithm to estimate Shapley values [3]. The algorithm is open source and available on GitHub, with the website located at https://github.com/shap/shap.
The following text will briefly describe how Kernel SHAP estimates Shapley values.

A principle of Shapley values

1. The Shapley value possesses the following property: the sum of contributions from all participants equals the total payoff of the grand coalition ***F***. Assuming the gain function is represented by v, this property is expressed by Equation 15 [2,4].

$$v(\boldsymbol{F}) = \sum_{i=1}^{|\boldsymbol{F}|}\varphi_i \quad \text{(Eq.15)}$$

Here, $|\boldsymbol{F}|$ represents the number of participants, and $v(\boldsymbol{F})$ denotes the total gain from all participants.

2. The gain for the coalition ***F*** is represented by Equation 16.

$$v(\boldsymbol{F}) = v(\{x_1, x_2, \ldots, x_m\}) = f(\vec{x}) - E[f(\vec{x})] \quad \text{(Eq.16)}$$

By deducing from Equations 15 and 16, and setting $E[f(\vec{x})] = \varphi_0$, then can obtain Equation 19.

$$f(\vec{x}) = \varphi_0 + \sum_{i=1}^{|\boldsymbol{F}|}\varphi_i \quad \text{(Eq.17)}$$

This formula is referred to as the additive feature attribution of Shapley values [5]. φ_i represents the Shapley value of the *i*th feature. This formula indicates that Shapley values can be transformed into a linear equation, where the features are additive.

LIME

The core idea of the LIME algorithm is to use a simple model to explain a complex model [6]. The algorithm

consists of three steps: the first step involves simplifying the original features to obtain a simplified feature vector; the second step perturbs the simplified feature vector; the third step involves training a simple linear model g (such as linear regression) using the perturbed simplified features; the fourth step transforms the perturbed simplified features back to the original feature format and applies them to the original function f for evaluation [6]. If $g(\vec{z}') \approx f(\vec{z})$, it can be considered that the linear model g provides a good explanation for the original model f.

The following text will provide a detailed description of the calculation process for each step.

Step one involves simplifying the original features to obtain a simplified feature vector. For the model f in Equation 5, a set of simplified input features can be created to indicate whether a feature is present in the input feature vector of function f. This simplified input vector is represented as per Equation 18.

$$\vec{x}' = [x_1' \ x_2' \ \dots \ x_m'] \quad \text{(Eq.18)}$$

x'_j is a binary variable indicating whether the corresponding feature x_j in the feature vector $\vec{x}$ is observed (1 if observed, 0 otherwise). For example, if the feature vector is:

$$\vec{x} = [1\ 2\ 3\ NA]$$

Then:

$$\vec{x}' = [1\ 1\ 1\ 0]$$

Additionally, for the aforementioned calculations, it can be assumed that there exists a mapping function h that maps $\vec{x}'$ to $\vec{x}$, and this function is represented as per Equation 19.

$$h(\vec{x}') = \vec{x} \quad \text{(Eq.19)}$$

Step two involves perturbing the simplified feature vector. Given that $\vec{x}' = [1\ 1\ 1\ 0]$, the vector can be perturbed to obtain $\vec{z}'$. The values of $\vec{z}'$ after perturbation are as follows:

$$\vec{z}' = [1\ 0\ 1\ 0]$$

In simple terms, after perturbation, $\vec{z}'$ corresponds to observable features, namely the first feature x_1 and the third feature x_3. It is important to note that the perturbed $\vec{z}'$ should be close to $\vec{x}'$, that is, $\vec{z}' \approx \vec{x}'$.

Step three involves substituting the obtained $\vec{z}'$ into Equation 19, which allows the mapping of $\vec{z}'$ to $\vec{z}$.

$$\vec{z} = h(\vec{z}') = [x_1\ NA\ x_3\ NA]$$

Subsequently, a linear regression model g is trained using $\vec{z}'$, and $\vec{z}$ is applied to the original function f. When $g(\vec{z}') \approx f(\vec{z})$ holds, and $\vec{z}' \approx \vec{x}'$ after perturbation, it can be considered that the model g provides a good explanation for f.

LIME defines a loss function $L(f, g, \pi_x)$ such that when $\vec{z}'$ is very close to $\vec{x}'$, the loss function aims for $g(\vec{z}')$ to be very close to $f(\vec{z})$. π_x is a measure of the distance between $\vec{x}'$ and $\vec{z}'$, and when the distance is large, π_x plays a penalizing role in the loss function [6]. Additionally, LIME provides a function $\Omega(g)$ to describe the complexity of the model g [6]. Therefore, the ultimate goal of LIME is to find a function g that minimizes the objective function, as shown in Equation 20.

$$\operatorname{argmin}\big(L(f, g, \pi_x) + \Omega(g)\big) \quad \text{(Eq.20)}$$

Kernal Shap

Through Equation 17 and LIME, it can be inferred that if the function $g(\vec{z}')$ represents a linear explanatory model found for f, then when all elements are present in $\vec{z}'$ (all elements in $\vec{z}'$ are 1), its mathematical expression is given by Equation 21.

$$g(\vec{z}') \approx f(\vec{z}) = \varphi_0 + \textstyle\sum_{i=1}^{|F|} \varphi_i \quad \text{(Eq.21)}$$

Through Equation 21, it is evident that as long as a linear function for $g(\vec{z}')$ is identified, estimates for the Shapley values of each feature can be obtained. Therefore, Kernel SHAP identifies the most suitable g for Shapley value estimation by minimizing Equation 20, where the computational speed of Kernel SHAP is faster than the direct computation speed of Shapley values [3]. As this section does not focus on the optimization process of Kernel SHAP but rather highlights its capability to estimate Shapley values, specific details of the optimization process will not be further described.

In this study, a neural network is employed as the explanatory function for LIME to estimate Shapley values. Specifically, the trained neural network f is incorporated into the Shap framework to obtain estimates by fitting g.

SUPPLEMENTARY REFERENCES

1. Goodfellow I, Bengio Y, Courville A. 6.2. 2.3 Softmax units for multinoulli output distributions. Deep learning. 2016; 180.

2. Roth AE. The Shapley value: essays in honor of Lloyd S. Shapley; Cambridge University Press: Cambridge. 1988.

3. Lundberg SM, Lee SI. A unified approach to interpreting model predictions. Advances in neural information processing systems. 2017; 30.

4. Winter E. The shapley value. Handbook of game theory with economic applications. 2002; 3:2025–54.

5. Lipovetsky S, Conklin M. Analysis of regression in game theory approach. Applied Stochastic Models in Business Industry. 2001; 17:319–30.

6. Ribeiro MT, Singh S, Guestrin C. "Why should i trust you?" Explaining the predictions of any classifier. In Proceedings of the Proceedings of the 22nd ACM SIGKDD international conference on knowledge discovery and data mining. 2016; 1135–44.
