# Supplementary material for: Sensitivity of substrate translocation in chaperone-mediated autophagy to Alzheimer’s disease progression
Source: Aging (Albany NY). 2024 May 23;16(10):9072–105. doi: 10.18632/aging.205856 (PMC11164475; doi:10.18632/aging.205856)
Supplement: Supplementary Figures [file aging-16-205856-s002.pdf]

SUPPLEMENTARY FIGURES

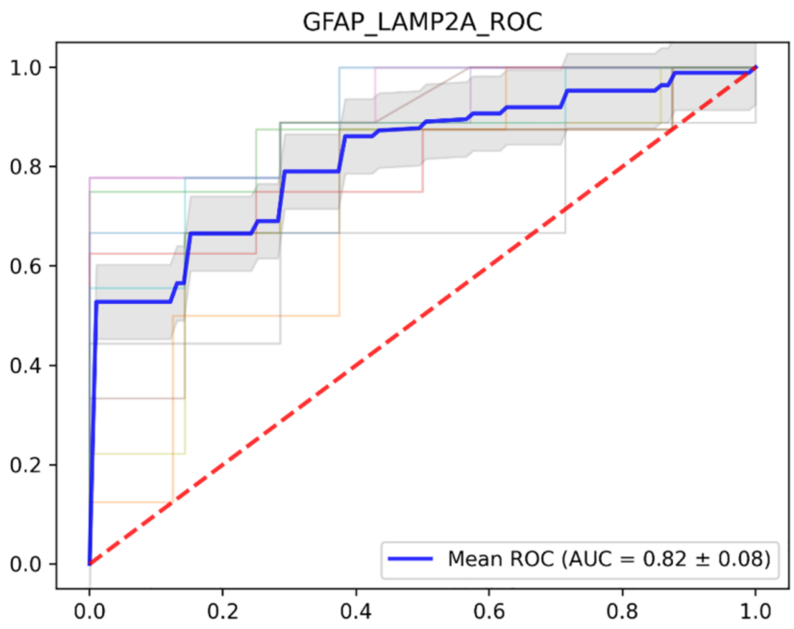

Supplementary Figure 1. Results of ten-fold cross validation of support vector machine model in GFAP and LAMP2A combination.

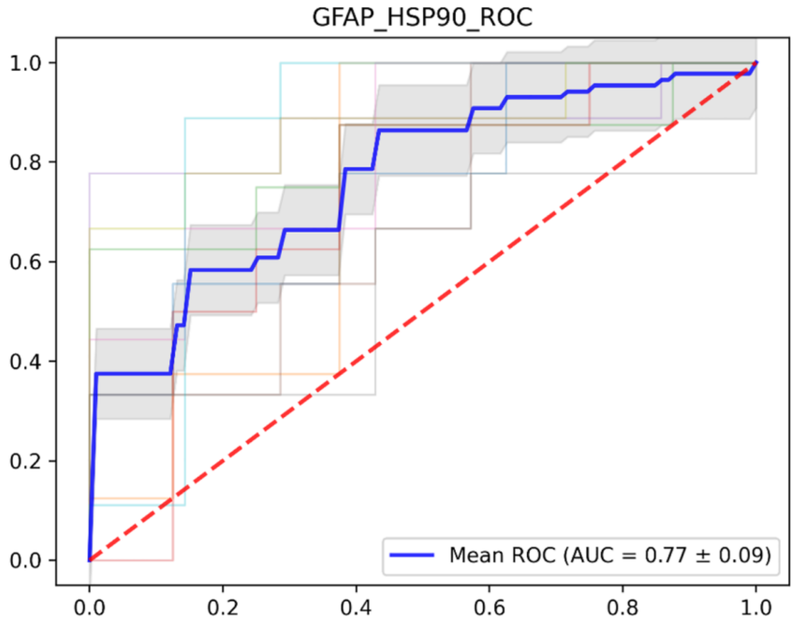

Supplementary Figure 2. Results of ten-fold cross validation of support vector machine model in GFAP and HSP90AB1 combination.

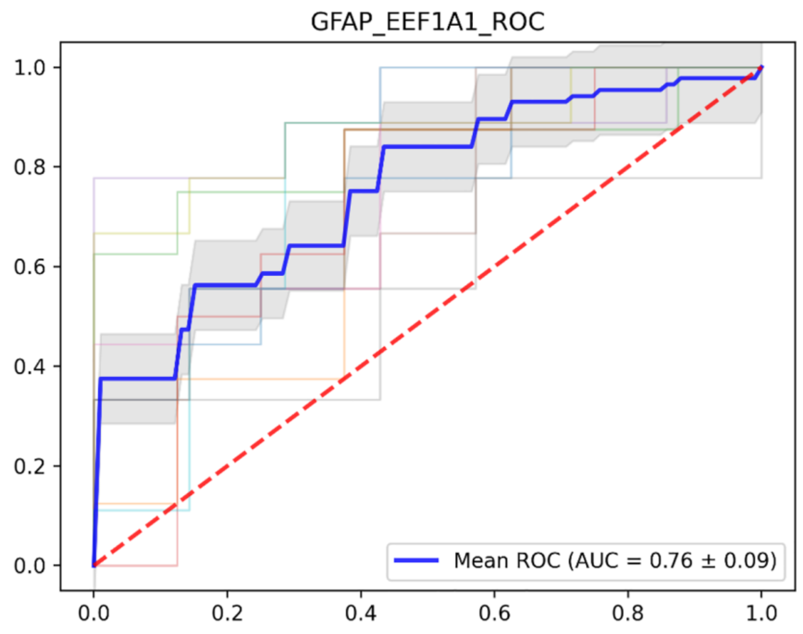

Supplementary Figure 3. Results of ten-fold cross validation of support vector machine model in GFAP and EEF1A1 combination.
